# Antioxidant activity of mesenchymal stem cell-derived extracellular vesicles restores hippocampal neurons following seizure damage

**DOI:** 10.7150/thno.58632

**Published:** 2021-04-03

**Authors:** Qiang Luo, Panpan Xian, Tian Wang, Shengxi Wu, Tangna Sun, Wenting Wang, Bo Wang, Hao Yang, Yanping Yang, Han Wang, Weiping Liu, Qianfa Long

**Affiliations:** 1 Mini-invasive Neurosurgery and Translational Medical Center, Xi'an Central Hospital, Xi'an Jiaotong University. No. 161, West 5th Road, Xincheng District, Xi'an, 710003, P. R. China.; 2 Department of Neurobiology, School of Basic Medicine, Fourth Military Medical University, No. 169 Changle West Road, Xi'an, 710032, P. R. China.; 3 Department of Neurosurgery, Xijing Hospital, Fourth Military Medical University, No. 127 Changle West Road, Xi'an, 710032, P. R. China.; 4 College of Medicine, Yan'an University, Yongxiang Road, Baota District, Yan'an, 716000, China.

**Keywords:** extracellular vesicles, hippocampal neuron, mesenchymal stem cells, oxidative stress, seizures

## Abstract

Oxidative stress is a critical event in neuronal damage following seizures. Mesenchymal stem cell-derived extracellular vesicles (MSC-EVs) have been shown to be promising nanotherapeutic agents in neurological disorders. However, the mechanism underlying MSC-EVs therapeutic efficacy for oxidative stress-induced neuronal damage remains poorly understood.

**Methods:** We investigated the antioxidant and restoration activities of MSC-EVs on hippocampal neurons in response to H_2_O_2_ stimulation *in vitro* and seizures *in vivo*. We also explored the potential underlying mechanism by injecting adeno-associated virus (AAV)-nuclear factor erythroid-derived 2, like 2 (Nrf2), a key antioxidant mediator, in animal models.

**Results:** MSC-EVs were enriched in antioxidant miRNAs and exhibited remarkable antioxidant activity evident by increased ferric ion-reducing antioxidant ability, catalase, superoxide dismutase, and glutathione peroxidase activities and decreased reactive oxygen species (ROS) generation, DNA/lipid/protein oxidation, and stress-associated molecular patterns in cultured cells and mouse models. Notably, EV administration exerted restorative effects on the hippocampal neuronal structure and associated functional impairments, including dendritic spine alterations, electrophysiological disturbances, calcium transients, mitochondrial changes, and cognitive decline after oxidative stress *in vitro* or *in vivo*. Mechanistically, we found that the Nrf2 signaling pathway was involved in the restorative effect of EV therapy against oxidative neuronal damage, while AAV-Nrf2 injection attenuated the antioxidant activity of MSC-EVs on the seizure-induced hippocampal injury.

**Conclusions:** We have shown that MSC-EVs facilitate the reconstruction of hippocampal neurons associated with the Nrf2 defense system in response to oxidative insults. Our study highlights the clinical value of EV-therapy in neurological disorders such as seizures.

## Introduction

Cell-based therapy has shown great promise in neurological diseases. However, multiple stem cell translational studies have shown that these approaches are limited by their safety, ethical issues, or national legislation 1. Mesenchymal stem cells (MSCs) are characterized by low immunogenicity, immunomodulatory properties, ability to expand in culture, and ease of isolation 2, making them an attractive stem cell type better suited for clinical use. Increasing evidence suggests that extracellular vesicles (EVs) are more effective than their parental cells (*e.g.,* MSCs) as restorative therapy in neurological disorders 3. Notably, EVs contain functional cargos and have been associated with the pathogenesis, progression, and prognosis of various diseases through their roles in immunomodulation, metabolic regulation, and signal transduction 4. Furthermore, biological characteristics, including the ability to cross the blood-brain barrier and resistance to freezing and thawing, favor their use in neurological deficits 5. Previous studies, including ours, have reported that MSC-EVs can serve as a cell-free therapeutic agent in treating multiple neurological diseases 3, 6 and can target neuronal cells in different brain pathologies 6, 7. However, the precise role of MSC-EVs in preventing neuronal damage remains poorly understood.

Seizures affect more than 70 million people with epilepsy, traumatic brain injury, and stroke worldwide 8. Hippocampal neurons are highly susceptible to seizure-induced damage and perturbations of neuronal structure and function contribute to epileptogenesis 9. Several studies have shown that a decrease in hippocampal inhibition or loss of hippocampal neurons results in spatial disruptions, recurrent seizures, and memory impairments 10, 11. Interestingly, oxidative stress, a significant pathophysiological process in experimental epileptogenesis, has been observed in human epilepsy 12, 13. Exposure of epileptogenic hippocampal neurons to oxidative stress can cause neuronal apoptosis, cell loss, mitochondrial dysfunction, and electrophysiological disturbances; therefore, targeting oxidative stress in the hippocampus can improve disease outcomes 12, 14.

Nuclear factor (erythroid-derived 2)-like 2 (Nrf2) is among the multiple molecular patterns associated with antioxidation, and Nrf2-targeted therapies exert neuroprotective effects in epilepsy and other neurological disorders in response to oxidative stress 15. Nrf2 is retained in the cytoplasm by Kelch-like ECH-associated protein 1 (Keap1) 16, which coordinates the expression of various target genes, such as *NAD(P)H quinone oxidoreductase 1* (*NQO1*) and *heme oxygenase-1* (*HO-1*), that encode antioxidant mediators, resulting in an orchestrated protective response to hippocampal neuron damage in seizures 17. Here, we determined that MSC-EVs exerted a strong antioxidant effect on the primary culture of hippocampal neurons in response to stress and were enriched with antioxidative microRNAs (miRNAs). Thus, we hypothesized that the antioxidant activity of MSC-EVs might facilitate the restoration of hippocampal neurons, and the Nrf2 defense system might be involved in the protective effect of EV therapy in seizures.

We used MSC-EVs to treat H_2_O_2_-stimulated primary cultures of hippocampal neurons and pilocarpine-induced seizures in wild-type and adeno-associated virus (AAV)-injected mice (Figure S1). Our data showed that MSC-EVs exerted antioxidant activity and restorative effects on hippocampal neuronal cells in response to oxidative insult and the Nrf2 signaling pathway was involved in the antioxidant potential of MSC-EVs in seizures. These results suggested that the antioxidant activity of MSC-EVs facilitates the reconstruction of hippocampal neurons following seizures, underscoring the value of EV nanotherapy in neurological diseases associated with oxidative damage.

## Materials and methods

### Animals

C57BL/6 mice, pregnant females (embryo at 16-18 days) and adult males (8-10-week-old), were purchased from the Experimental Animal Center of Xi'an Jiaotong University. All animal procedures were performed in accordance with the Guide for the Care and Use of Laboratory Animals (8^th^ edition, 2011) and approved by the Ethics Review Board of Xi'an Central Hospital, Xi'an Jiaotong University. Animals were housed in a controlled environment with a 12:12-hour light/dark cycle with food and water provided *ad libitum*.

### Isolation and characterization of MSC-EVs

Consenting donors were selected from full-term puerperal women in good health, and MSCs were isolated from their umbilical cords as described in our previous report 18. The procedures were approved by the Ethics Committee of Xi'an Central Hospital (No. 20180715-1) and conducted in accordance with the guidelines of the National Institutes of Health, USA. For the preparation of allogeneic MSCs, the primary culture was obtained from enzymatic digestion of Wharton's Jelly and expanded using the complete culture medium (CCM) consisting of Dulbecco's Modified Eagle's Medium (DMEM) and 16.5% fetal bovine serum (FBS). Primary antibodies against CD166, CD105, CD90, CD45, CD34, and CD11b (Table S5) were used to detect the surface antigens on MSCs as the minimum criteria established by the Mesenchymal and Tissue Stem Cell Committee of International Society for Cellular Therapy. Multi-potency of MSCs was tested using the StemPro® osteogenesis, chondrogenesis, and adipogenesis (Gibco, Maryland, USA) differentiation kits according to the manufacturer's instructions.

EV-depleted FBS was harvested by ultracentrifugation of FBS (10099-141C, Gibco, USA) at 100,000 ×g for 16 h 19. MSCs at 70% confluency (5^th^ passage) were washed with PBS twice and cultured in DMEM/F12 containing 10% EV-depleted FBS for 48 hours (h). Subsequently, the supernatant was collected and processed for EV isolation as described previously 18. Identification of EVs was performed using Western blotting to probe for the representative proteins CD81 (10630D, Invitrogen, USA), CD63 (10628D, Invitrogen, USA), CD9 (ab92726, Abcam, USA), and TSG101 (ab125011, Abcam, USA). The morphology and size distribution of EVs were examined by TEM (JEM-1400, JEOL Ltd., Japan) and NTA (ZetaVIEW S/N 17-310, PARTICLE METRIX, Germany) as previously described 6, 18.

### Hippocampal neuron preparation and transfection

Hippocampi were dissected from embryonic day 16-18 mice in a sterile environment, and the tissues were digested using 0.125% trypsin for 10 minutes at 37°C, followed by washing with DMEM containing 10% FBS to stop the digestion. The cells were then cultured in the neurobasal medium (21103-049, Gibco, USA) supplemented with 2% B27 (17504-044, Gibco, USA), 100 U/mL penicillin-streptomycin (15140-122, Gibco, USA), and 2 mM L-glutamine (25030081, Gibco, USA) with the culture medium change every 3 days. Viable cells were determined by trypan blue exclusion and counted using an Automated Cell Counter (JSY-SC-031, BodBoge, China). Hippocampal neurons at over 98% confluence were used in the following study.

Various miRNAs (miR-215-5p, miR-424-5p, miR-31-3p, miR-193b-3p and miR-200b-3p), NC, NC-FAM, inhibitors, and mimics were designed by GenePharma (Shanghai, China); the miRNA sequences are shown in Table S4. Target miRNAs were transfected using the Lipofectamine 3000 kit based on the manufacturer's instructions. Briefly, 5 μg target miRNAs and 10 μL Lipofectamine 3000 were diluted in DMEM, added to the cell culture wells, and incubated at 37 °C with 5% CO_2_. After 6 h, the medium was replaced and the cells were collected for subsequent experiments after another 48h incubation. The transfection of hippocampal neurons was performed in NC, NC+H_2_O_2_, inhibitor, and mimic groups.

### AAV injection in mice

For knockdown of Nrf2 in the hippocampus of mice, the hippocampi were injected with AAV-pAKD-CMV-bGlobin-mCherry-H1-shRNA (*Nfe2l2*) or pAKD-CMV-bGlobin-mCherry-H1-shRNA (NC) (OBiO Technology Corp., Ltd., Shanghai, China) 21 days before seizure induction according to the manufacturer's instructions. In brief, the mice were injected with a dosage of 4.5 × 10^6^ V.G. AAV-*Nfe2l2* or AAV-NC by using a 0.5 μl microsyringe (Hamilton, Reno, NV) at a rate of 0.05 μl per min, into the CA1 region pyramidal cell layer (2.1 mm posterior to bregma, 2.3 mm lateral to the midline, at a depth of ∼2 mm from the brain surface). To prevent reflux and ensure complete dispersion of the AAV, the syringe was removed at least 15 min after the injection. The efficiency of *Nrf2* knockdown in hippocampi was estimated by mCherry fluorescence (Figure 8A) and Western blotting (Figure 8B). AAV-shRNA (*Nfe2l2*)-treated and AAV-shRNA (NC)-treated mice were designated as the AAV-Nrf2 and AAV-NC groups, respectively.

### Model establishment and EV treatment

Oxidative stress in primary culture hippocampal neurons was induced by H_2_O_2_ stimulation as described 20. In brief, hippocampal neurons were cultured in 96-well plates at a density of 5 × 10^3^ cells per well in the neurobasal medium for 7 days. Subsequently, the medium was replaced with H_2_O_2_ at different concentrations (20 μM, 50 μM, 80 μM, 100 μM, 150 μM, 200 μM, 250 μM, 300 μM, 400 μM, and 500 μM) in the neurobasal medium for 3 h. Thereafter, the hippocampal culture medium was replaced with incremental concentrations of MSC-EVs (5 μg/mL, 10 μg/mL, 20 μg/mL, and 30 μg/mL) and incubated in 5% CO_2_ at 37 °C for 24 h. Cell viability was detected by the Cell Counting Kit-8 (CCK-8) (Beyotime, Beijing, China) assay and estimated by measuring optical density (OD) using a microplate reader (Bio-Rad Laboratories Inc., Hercules, CA, USA) at 450 nm. The appropriate concentration of H_2_O_2_ was selected at the cell viability reduced by 50% in response to stimulation. The predefined dose of MSC-EVs and an equivalent volume of PBS were used to treat H_2_O_2_-stimulated hippocampal neurons as the H_2_O_2_+MSC-EVs group and H_2_O_2_+PBS group, respectively.

Intraperitoneal injection of 290~320 mg/kg pilocarpine hydrochloride (S4231, Selleck Chemicals, USA) was used to induce SE as previously described 6, 18, and mice showing consistent stage 4-5 seizures were administered diazepam injections (10 mg/kg) 2 h after onset to terminate the seizures. The animals that did not show consistent acute seizure activity (stage 0-3) or developed severe tonic-clonic seizures were excluded from the study. The seizure models were intravenously injected with MSC-EVs and PBS as the SE+MSC-EVs and SE+PBS groups, respectively. The dose response was examined by antioxidant response assays; 50 μg MSC-EVs diluted in 150 μL sterile PBS was used to treat seizure mice in this study (Figure S6). The normal mice received the same volume of PBS as the Sham group.

### Antioxidant activity test

The antioxidant activity of MSC-EVs on H_2_O_2_-stimulated primary cultures of hippocampal neurons was tested according to the procedures described in our recent study 21. The cell supernatant in each group (n = 6~9 samples per group) was collected by centrifugation at 12000 *g* for 5 min, and the samples and reaction reagents, including those from the ferric ion reducing antioxidant power (FRAP) (A015-3, Nanjing Jiancheng Bioengineering Institute, China), catalase (CAT) (S0051, Beyotime, China), glutathione peroxidase (GSH-PX) (A005, Nanjing Jiancheng Bioengineering Institute, China) and superoxide dismutase (SOD) (A001-3, Nanjing Jiancheng Bioengineering Institute, China) kits, were added to 96-well flat-bottom plates. The antioxidant effect or enzyme activity was detected using a microplate reader (Bio-Rad Laboratories Inc., Hercules, CA, USA) per the manufacturer's instructions. Each test was replicated 3 times.

### Flow cytometry

Hippocampal neuronal cells were seeded onto 6-well plates at a density of 3 × 10^5^ cells per well. After treatment with H_2_O_2_ and MSC-EVs in the experimental groups (n = 4~5 per group), the cells were collected in EP tubes and stained with 10 μM 2′,7′-dichlorodihydrofluoroscein diacetate (D6883, Sigma-Aldrich, CA, USA) to detect ROS generation or Annexin-V/PI (556547, BD, USA) to probe cell apoptosis. At least 5 cell culture samples were evaluated on a FACS Calibur instrument (FACS101, Becton Dickinson, USA), and the data were analyzed using Cell Quest software (Becton Dickinson, USA).

### MiRNA sequencing to determine the exosomal composition

To detect the antioxidant cargoes in MSC-EVs, MSCs were preconditioned with H_2_O_2_ 22, 23 in FBS-depleted media, and EVs were isolated as described above. Total RNA from MSC-EVs (Control group, n = 3) and H_2_O_2_-preconditioned MSC-EVs (H_2_O_2_ group, n = 3) was extracted using the miRNeasy Serum/Plasma Kit (217184, Qiagen, Germany), and the RNA integrity and concentration were examined by the Agilent 2100 Bioanalyzer (Agilent Technology, USA) and NanoDrop 2000 (Thermo Fisher, USA). Subsequently, the small RNA libraries were generated using 1 µg of total RNA based on the NEBNext Multiplex Small RNA Library Prep Set from Illumina (E7580S, NEB, USA) per the manufacturer's instructions. The quality of the cDNA library was assessed using a DNA chip on the Agilent 2100 Bioanalyzer. Finally, the library was sorted using the Illumina HiSep X Ten platform. The sequencing and analysis of small RNA were performed by Biotechnology Corporation (Shanghai, China).

### ELISA

Culture supernatant (n = 5 per group) and hippocampal samples (n = 5~6 per group) in the experimental groups were prepared as recently described 21 and then analyzed using a mouse ELISA kit for 8-OHdG (CEA660Ge, Cloud-Clone, China) according to the manufacturer's instructions. The absorbance was measured with a microplate reader (Bio-Rad Laboratories Inc., Hercules, CA, USA) at 450 nm. Each measurement was performed in triplicate.

### Calcium imaging

The calcium indicator Fluo-8 AM (ab142773, Abcam, CA, USA) was used to detect the intracellular Ca^2+^ oscillations in each group of primary culture cells, and the procedures were performed as previously described 18, 24. The cultures were washed in media containing 4 μM Fluo-8 AM for 20 min at 37°C and 5% CO_2_ to load the dye into the hippocampal neurons. Next, the samples were washed three times and stored in artificial cerebrospinal fluid (ACSF, containing 124 mM NaCl, 25 mM NaHCO_3_, 2.5 mM KCl, 1 mM KH_2_PO_4_, 2 mM CaCl_2_, 2 mM MgSO_4_, and 10 mM glucose). After resting Ca^2+^ levels were recorded in ACSF for 20 seconds, 10 μM adenosine monophosphate (ATP) was used to stimulate Ca^2+^ influx. Fluorescent signals were excited at a wavelength of 488 nm and imaged every 1 second for 120 seconds using a confocal microscope (Olympus, FV3000, Japan). Calcium influx and resting Ca^2+^ levels were measured in individual hippocampal neurons using the image analysis software Cellcens (Olympus, Japan). More than 100 cells for each experimental condition (ΔF/F) were analyzed using Igor Pro software (WaveMetrics, Oregon, USA), and the results from at least three independent experiments were averaged.

### JC-1 staining

The change in the mitochondrial membrane potential (MMP) in the primary culture of hippocampal neurons was measured using JC-1 (Solarbio, 3520-43-2, Beijing, China) staining, a dual-emission membrane potential-sensitive probe that exists as a green fluorescent monomer at a low MMP and forms aggregates with a fluorescent shift from green to red at a high MMP 25. Cell samples, including the control, H_2_O_2_+PBS, and H_2_O_2_+MSC-EVs groups (n = 5 per group), cultured in confocal dishes, were washed three times with PBS, after which 5 mM JC-1 was added to the culture and incubated for 30 min at 37 °C and 5% CO_2_. The change in fluorescence at 488 nm (green, excitation) and 594 nm (red, emission) was monitored by a confocal microscope (Olympus, FV3000, Japan). Data were processed using Image-Pro Plus V 6.0 (Bethesda, Maryland, USA), and the results are represented as the average ratio of red to green fluorescence intensity. Each determination was an average of at least 3 independent experiments.

### Whole-cell patch-clamp recording

Primary cultures of hippocampal neurons (n = 8) and hippocampal coronal slices (n = 6~9 cells per group) (300 μm, Bregma: 1.3-0.5 mm) in each group were prepared as described in our previous reports 24, 26. The cultures or slices were placed in a recording chamber and constantly perfused with carbogenated ACSF at 25-28 °C (TC-324B, Warner Instruments, USA). Recording micropipettes (BF150-86-7.5, Sutter Instruments, USA) were pulled in a horizontal pipette puller (P-97, Sutter Instruments, USA) with a tip resistance of 3-6 MΩ. Patch pipettes were filled with the internal solution (130 mM K-gluconate, 1 mM ethylene glycol bis (2-aminoethyl ether) tetraacetic acid (EGTA), 2 mM Mg-ATP, 0.3 mM Na-guanosine 5′-triphosphate, 5 mM Na-phosphocreatine and 10 mM hydroxyethyl piperazine ethane sulfonic acid (HEPES) (pH 7.3)). Either currents or membrane potential recordings were made using an Axopatch 200B amplifier (Axon Instruments, Molecular Devices, USA). Signals were filtered at 2 kHz and sampled at 5 kHz with a Digidata 1322A and Clampex 9.0 (Molecular Devices, USA). The data were stored on a computer and analyzed using pClamp 10.6 software (Molecular Devices, CA, USA).

### Golgi staining

Golgi staining was performed as in our previously described study with minor modifications 27. Animals (n = 5 per group) were perfused with 0.9% saline solution or 0.01 M PBS (pH 7.4). The brain tissue was removed and immersed in Golgi-Cox solution (consisting of 5% potassium chromate, 5% potassium dichromate, and 5% mercuric chloride) for further fixation, after which the tissue was maintained in the dark at room temperature for 2-3 days. Then, the brains were transferred to fresh Golgi-Cox Solution for an additional 7 days. Coronal sections (200 µm) were cut serially. For staining, brain sections were washed in deionized water for 5 min 2 times, placed in 50% NH4OH for 5 min, and washed in deionized water for 5 min 2 times. The sections were then incubated in 5% sodium thiosulfate for 10 min. After rinsing with PBS, dehydration with gradient ethanol was performed. Finally, the sections were mounted and observed under the bright field of confocal microscope FV1000, images were taken by z-stack scanning with an excitation wavelength of 405 nm, and then the virtual color was converted into green color.

### Immunochemistry

Hippocampal neuronal samples (n = 5 per group) and brain sections (n = 5~6 per group) through the hippocampus (bregma from -2.64 mm to -3.48 mm) were selected and processed for immunostaining as previously described 6, 18. Briefly, the cell and tissue samples were treated for 30 min in PBS solution containing 0.1% Triton-X 100 and an appropriate serum (10%) concentration selected based on the species in which the chosen secondary antibody was raised. Primary antibodies against 8-OHdG (1:200, Millipore, AB5830, USA), NeuN (1:1000, Millipore, ABN78, USA), Doublecortin (DCX, 1:200, Santa, sc-271390, USA), NeuN (1:1000, Abcam, ab104224, USA), iNOS (1:100, Proteintech, 18985-1-AP, USA), and Nrf2 (1:100, Abcam, ab31163, USA). After overnight incubation with the respective primary antibody solution, the cell and tissue samples were washed three times in PBS and subjected to the appropriate secondary antibody solution for 2 h at room temperature. Alexa Fluor 488-conjugated donkey anti-mouse IgG (H+L) (1:200, Invitrogen, A-21202, USA), Alexa Fluor 488-conjugated donkey anti-rabbit IgG (H+L) (1:200, Invitrogen, A-21206, USA), Alexa Fluor-conjugated 488 donkey anti-goat IgG (H+L) (1:200, Invitrogen, A-11055, USA), Alexa Fluor 594-conjugated donkey anti-mouse IgG (H+L) (1:200, Invitrogen, A-21203, USA), and Alexa Fluor 594-conjugated donkey anti-rabbit IgG (H+L) (1:200, Invitrogen, A-21207, USA) were used for the study. 4',6-Diamidino-2-phenylindole (DAPI; D9542, Sigma-Aldrich, USA) was used to probe cell nuclei. Images were captured using a confocal microscope (Olympus, FV10-ASW, Japan). The images were analyzed using Image-Pro Plus V 6.0 (Bethesda, Maryland, USA).

### Western blotting

Cell (n = 4~5 per group) and tissue (n = 5 per group) samples were processed for Western blotting as previously described 18. Briefly, proteins were extracted using radioimmunoprecipitation assay (RIPA) lysis buffer (Beyotime, P0013B, Beijing, China) and quantified using a BCA assay. Normalized protein samples were subjected to sodium dodecyl sulfate-polyacrylamide gel electrophoresis and transferred to polyvinylidene fluoride (PVDF) membranes (Millipore, MA, USA). Membranes were blocked using 5% skim milk in Tris-buffered saline Tween (TBST) at room temperature for 1.5 h and incubated with primary antibodies against CD81 (1:1000), CD63 (1:1000), CD9 (1:800), TSG101 (1:800), Nrf2 (1:1000, Proteintech, 16396-1-AP, USA), HO-1 (1:1000, Proteintech, 10701-1-AP, Illinois, USA), Keap1 (1:1000, Proteintech, 10503-2-AP, Illinois, USA), 4-HNE (1:1000, Invitrogen, MA5-27570, USA), DT (1:1000, Invitrogen, MA5-27575, USA), iNOS (1:1000), HMGB1 (1:1000, Proteintech, 10829-1-AP, USA), TOM20 (1:5000, Proteintech, 11802-1-AP, USA), FIS1 (1:1000, Proteintech, 10956-1-AP, USA), COX Ⅳ (1:5000, Proteintech, 11242-1-AP, USA), AMPA (1: 2000, ab109450, Abcam, USA), Glut1 (1:1000, Proteintech, 21829-1-AP, USA) and β-actin (1:100000, Abclonal, AC026, Wuhan, China) overnight at 4 °C, followed by washing with TBST three times. Membranes were then incubated with horseradish peroxidase-conjugated secondary antibodies (Thermo Fisher Scientific, New York, USA) at room temperature for 1.5 h. Labeled proteins were detected using a Bio-Rad imaging system (Bio-Rad, Hercules, CA, USA) and quantified using the Image Lab software package (Bio-Rad, CA, USA).

### Morris water maze (MWM) test

The learning and memory impairments in the Sham (n = 6), SE+PBS (n = 7), and SE+MSC-EV (n = 8) groups were assessed 3 months post-treatment using the MWM tracking system (SuperMaze, XRXM101, China). Each mouse was placed in water (23±1 °C) from any position (North, South, West, or East) of the MWM apparatus, and the time to reach a transparent platform located in the middle of the target quadrant was measured. Escape latency was recorded as 60 seconds if the mouse failed to find the platform. The mice received training sessions in the last 4 days (4 trials per day), during which the escape latency to find the platform was recorded as the parameter for analyzing the learning capacity. After 24 h from the last training session, the hidden platform was removed, and the percentage of the total time (%, PT) spent in the target quadrant and platform crossings (4 trials) was measured by Smart v2.5 software (SuperMaze, China) to determine the spatial memory impairments in each group.

### Statistical analysis

Data are expressed as the mean ± SEM. Multiple comparisons were analyzed using one-way analyses of variance (ANOVA) by the least significant difference test, and repeated measures ANOVA was carried out to analyze the differences in learning curves and traveling distance among the four groups after the Bonferroni post hoc test. The GraphPad Prism 7 software (GraphPad Prism, USA) was used to interpret the data. *P* values of less than 0.05 were considered to be statistically significant.

## Results

### Characterization of MSCs and MSC-derived EVs

MSCs, obtained from Wharton's jelly in the human umbilical cord as described in our recent reports 18, 21, showed characteristic features, such as high levels of stromal markers (Figure S2A) and multipotency 28. The MSC-derived vesicles were positive for classical exosomal markers, such as CD81, CD63, CD9, and TSG101 found on the surface of MSCs (Figure S2B). The nanoparticles displayed a typical cup-shaped morphology (Figure S2C) and an average diameter of 124.3 nm (Figure S2D), consistent with the typical characteristics of EVs according to the guidelines of the International Society for Extracellular Vesicles (2018).

### Antioxidative effect of MSC-EVs on H_2_O_2_-stimulated hippocampal neurons

**Figure 1:**
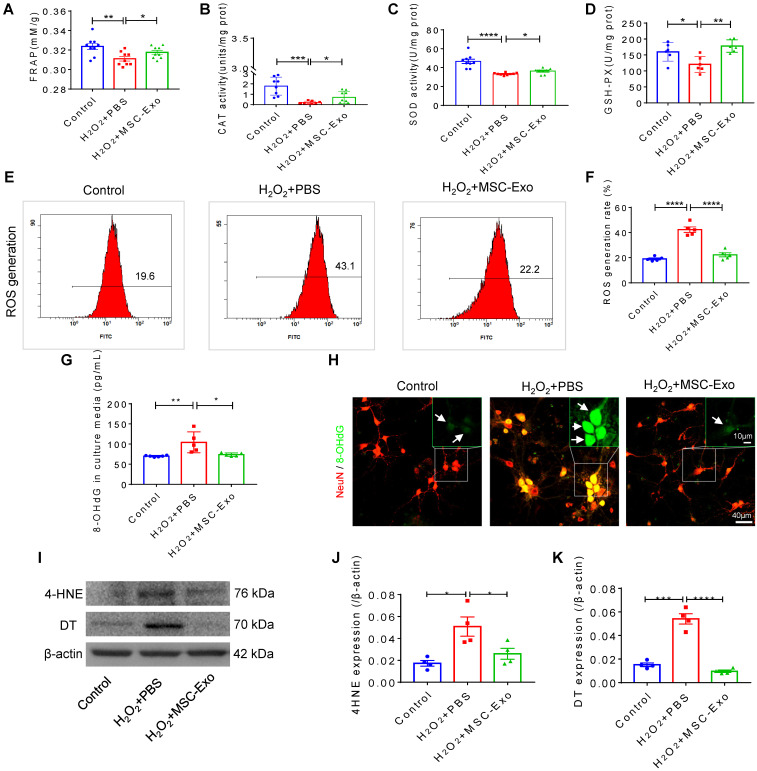
** Antioxidant potential of MSC-EVs on the primary culture of hippocampal neurons.** (A-D) Histograms show FRAP, CAT, SOD, and GSH-PX activities among experimental groups (n = 6~9 per group). (E-F) Flow cytometry analysis of the ROS generation in the primary culture of each group (n = 5). (G) ELISA test 8-OHdG for concentration in experimental groups (n = 5 per group). (H) Representative images of 8-OHdG (green) immunostaining with NeuN (red) in primary cultures of hippocampal neurons, green frames show magnified images of 8-OHdG (white arrow, green) in each group. Scale bar (H) = 40 µm, scale bar (magnified images) = 10 µm. (I) Western blots of 4-HNE and DT expression in hippocampal neurons. (J-K) Protein assay for the expression of 4-HNE (J) and DT (K) in the experimental groups (n = 4 per group). MSC-EVs: mesenchymal stem cell-derived extracellular vesicles; n: number; FRAP: Ferric ion reducing antioxidant power; CAT: catalase; SOD: superoxide dismutase; GSH-PX: glutathione peroxidase; ROS: reactive oxygen species; 4-HNE: 4-hydroxynoneal; 8-OHdG: 8-hydroxy-2 deoxyguanosin; DT: dityrosine. * *p* < 0.05, ** *p* < 0.01, *** p < 0.001, **** p < 0.0001.

The primary culture of hippocampal neurons was pretreated with 10 μg EVs (Figure S3) and subsequently stimulated by 100 μM H_2_O_2_ (Figure S4). MSC-EVs pretreatment increased FRAP (Figure 1A), CAT (Figure 1B), SOD (Figure 1C) and GSH-PX (Figure 1D) activity compared to the H_2_O_2_+PBS group (Figure 1A-D). Also, when ROS production in H_2_O_2_-stimulated hippocampal neurons was assessed by flow cytometry (Figure 1E), a reduction in the ROS generation rate (*vs*. H_2_O_2_+PBS) was detected in the H_2_O_2_+MSC-EVs group (Figure 1F). Moreover, our data showed that H_2_O_2_ stimulation resulted in elevated expression of 8-OHdG (DNA damage marker) (Figure 1G), 4-HNE (lipid peroxidation marker) (Figure 1J), and DT (protein oxidative marker) (Figure 1K), whereas EV treatment markedly decreased the expression of these markers (Figure 1I-K) compared to the PBS group. Additionally, immunostaining confirmed the DNA damage represented by 8-OHdG expression (Figure 1H) in the H_2_O_2_+PBS group, which was repaired by MSC-EV treatment. These results suggested that MSC-EVs exert strong antioxidant potential on the H_2_O_2_-stimulated hippocampal neurons.

### Presence of antioxidant miRNAs in MSC-EVs

**Figure 2:**
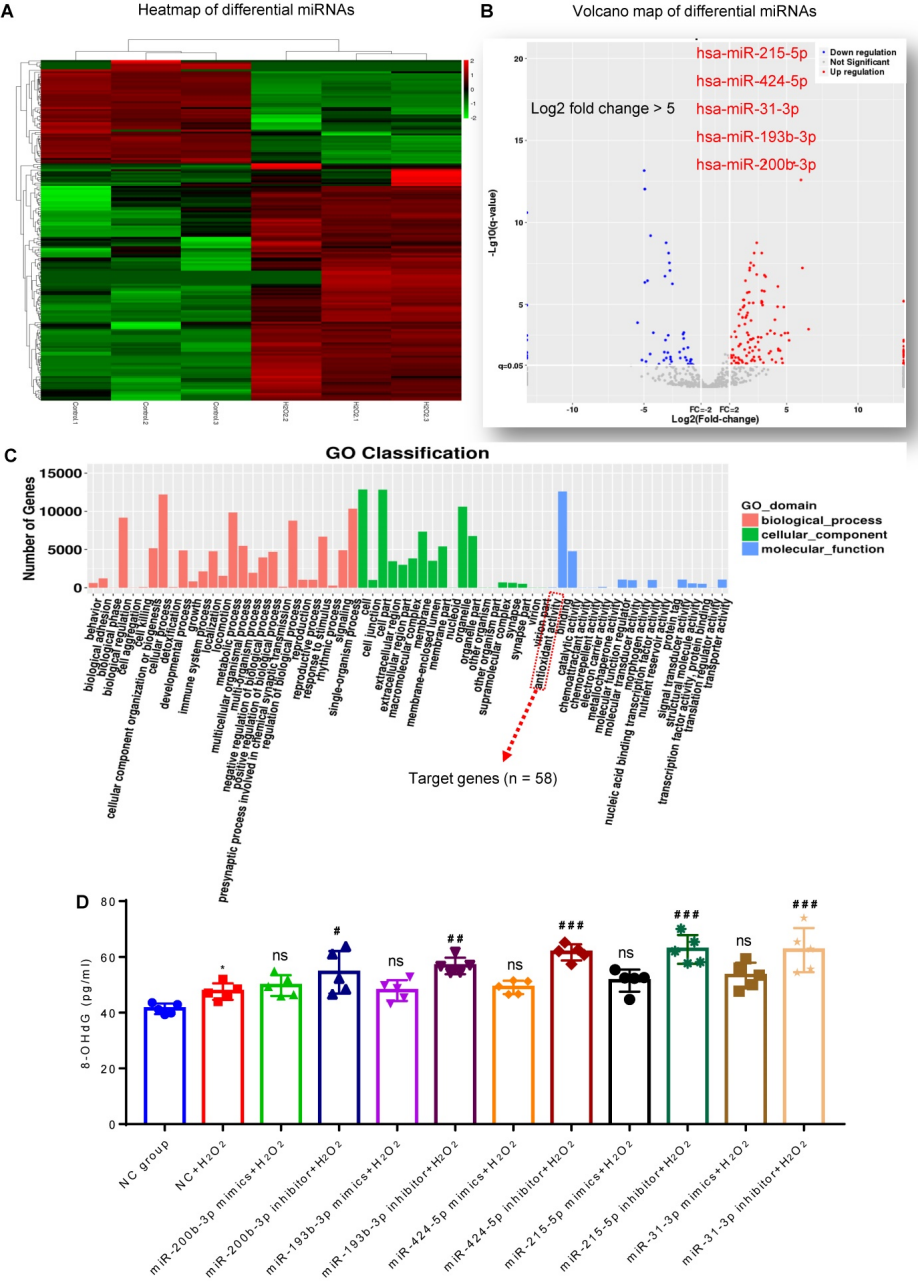
** Composition of antioxidant miRNAs in MSC-EVs.** (A) miRNA sequencing from MSC-EVs and conditioned EVs. (B) Volcano map of downregulated (blue) and up-regulated (red) miRNAs in conditioned EVs. (C) GO Classification of exosomal miRNA target genes (n = 58) associated with the antioxidant activity molecular function. (D) ELISA test for 8-OHdG generation in H2O2-stimulated hippocampal neurons after adding miRNA inhibitors and mimics (n = 5 per group). MSC-EVs: mesenchymal stem cell-derived extracellular vesicles; ns: no significance; n: number; GO: gene ontology; 8-OHdG: 8-hydroxy-2 deoxyguanosin. * *p* < 0.05 (vs. Control group), # *p* < 0.05 (vs. H_2_O_2_+PBS), ## *p* < 0.01 (vs. H_2_O_2_+PBS), ### *p* < 0.001 (vs. H_2_O_2_+PBS), ns *p* > 0.05 (vs. H_2_O_2_+PBS).

MiRNA sequencing was used to identify the cargos that might be responsible for the antioxidant potential of MSC-EVs. We compared the differentially expressed miRNAs between MSC-EVs and conditioned EVs (derived from H_2_O_2_-stimulated MSCs) 22 and found enrichment of specific miRNAs in MSC-EVs (Figure 2A and B; Table S1); differential expression analysis revealed upregulation of many miRNAs (log2-fold change > 5: miR-215-5p, miR-424-5p, miR-31-3p, miR-193b-3p and miR-200b-3p) in H_2_O_2_-conditioned EVs (Figure 2B). Gene Ontology (GO) classification (Figure 2C; Table S2) revealed that the exosomal miRNA target genes (n = 58) were associated with the antioxidant activity molecular function. MiRNA (miR-215-5p, miR-424-5p, miR-31-3p, miR-193b-3p, and miR-200b-3p) transfection showed that, compared to the NC+H_2_O_2_ group, miRNA inhibitors increased the concentration of 8-OHdG in oxidative stress-induced hippocampal neurons, while miRNA mimics did not alter the 8-OHdG concentration in the experimental groups (Figure 2D). The data implied that these miRNAs in MSC-EVs exerted the antioxidant potential on the H_2_O_2_-stimulated hippocampal neurons.

### MSC-EVs protected against oxidative stress-induced neuronal damage in primary culture

**Figure 3:**
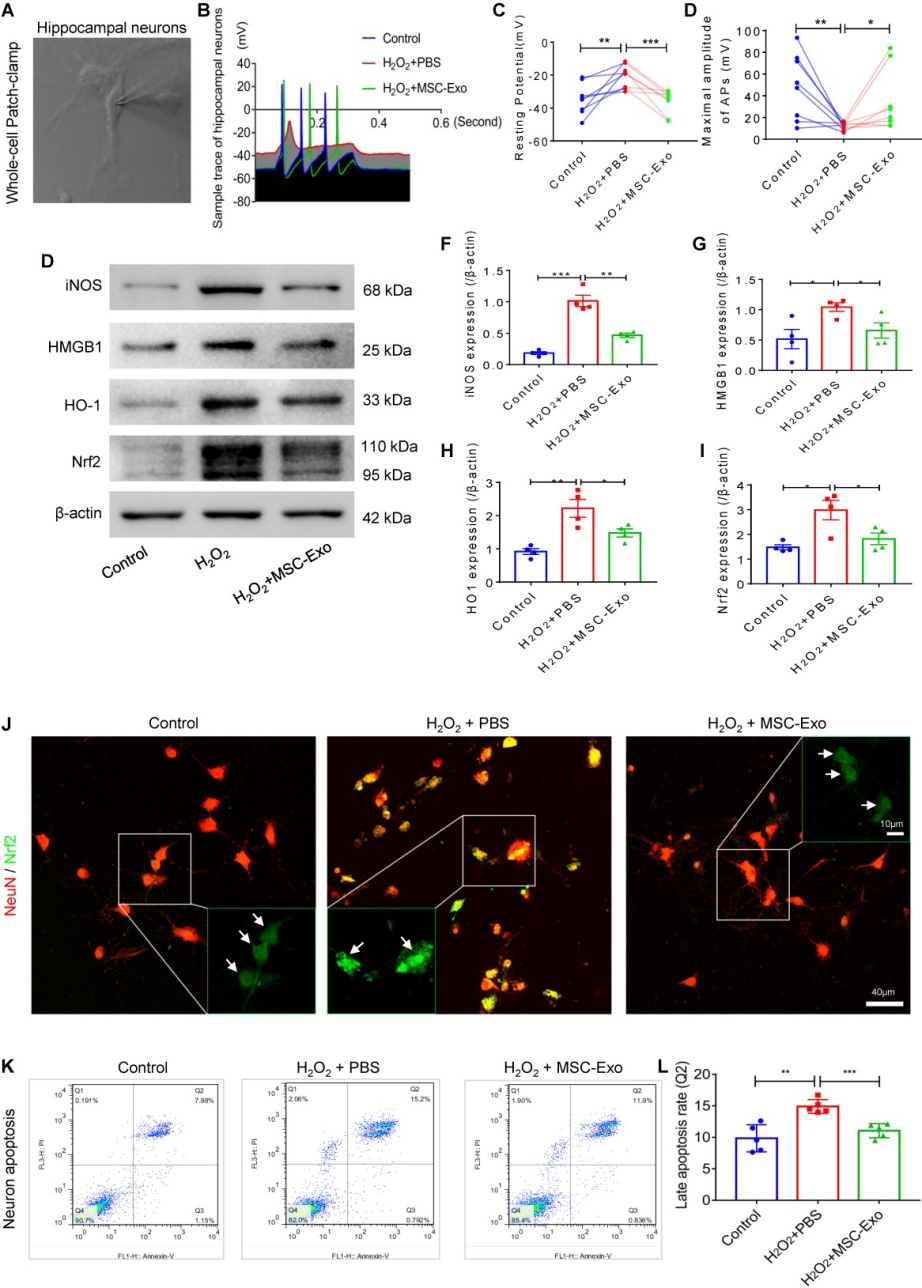
** Neuroprotection of MSC-EVs on H_2_O_2_-stimulated primary culture of hippocampal neurons.** (A) Whole-cell patch-clamp for the primary culture of hippocampal neuron. (B) Representative potential traces of hippocampal neurons in each group. (C-D) Summary data for the resting potential (C) and maximal amplitude of APs (D) in the primary culture obtained from each group (n = 8 per group). (E) Western blotting for stress-associated molecular patterns. (F-I) Histograms of the iNOS (F), HMGB1 (G), HO-1 (H), and Nrf2 (I) expression in the experimental groups (n = 4 per group). (J) Representative images of Nrf2 (green) immunostaining with NeuN (red) in the primary culture of hippocampal neurons; indicative nuclei translocation of Nrf2 (white arrow, green) is shown by magnified images (green frames) in the experimental groups. Scale bar (J) = 40 µm, scale bar (magnified images) = 10 µm. (K) Neuronal apoptosis in each group was detected by flow cytometry. (L) Histogram shows the late apoptosis rate of hippocampal neurons in response to H_2_O_2_ stimulation in the experimental groups (n = 4 per group). MSC-EVs: mesenchymal stem cell-derived extracellular vesicles; n: number; APs: action potentials; iNOS: inducible nitric oxide synthase; HMGB1: high mobility group box 1; HO-1: heme oxygenase-1; Nrf2: nuclear factor erythroid-derived 2, like 2. * *p* < 0.05, ** *p* < 0.01, *** *p* < 0.001.

Production of action potentials (APs) is the basic function of neurons; therefore, we first performed whole-cell patch-clamp experiments (Figure 3A) to detect the AP firing patterns (Figure 3B) in the culture of each group. The greater resting potential (hyperpolarization) (Figure 3C) and lower maximal amplitude of APs (mV) (Figure 3D) indicated electrophysiological damage in the hippocampal neurons in the H_2_O_2_ group compared to the control group. After MSC-EV treatment, these alterations in response to H_2_O_2_ stimulation (Figure 3C and D) were markedly reversed in the primary culture of hippocampal neurons. We also examined a series of stress-associated molecular patterns (SAMPs), including iNOS, HMGB1, HO-1, and Nrf2, by Western blotting (Figure 3E) or immunostaining (Figure 3J). The H_2_O_2_ stimulation-induced oxidative damage was identified by increased protein levels of iNOS (Figure 3F), HMGB1 (Figure 3G), HO-1 (Figure 3H), and Nrf2 (Figure 3I) in the PBS group. The stress change was also revealed by the nuclear translocation of Nrf2 (Figure 3J) in H_2_O_2_-stimulated hippocampal neurons. Notably, EV treatment significantly reduced the expression of these SAMPs (Figure 3F-I) and inhibited nuclear translocation of Nrf2 (green, Figure 3J) compared with PBS treatment. Next, neuronal apoptosis was detected by flow cytometry (Figure 3P), and the data showed that MSC-EV treatment significantly reduced the percentage of neuronal cells in the Q2 quadrant (representing late apoptosis) compared to H_2_O_2_ stimulation. These data suggested that MSC-EVs protected against oxidative stress-induced neuronal damage in the primary culture.

### MSC-EVs ameliorated H_2_O_2_-induced calcium transients and mitochondrial dysfunction in hippocampal neurons

**Figure 4:**
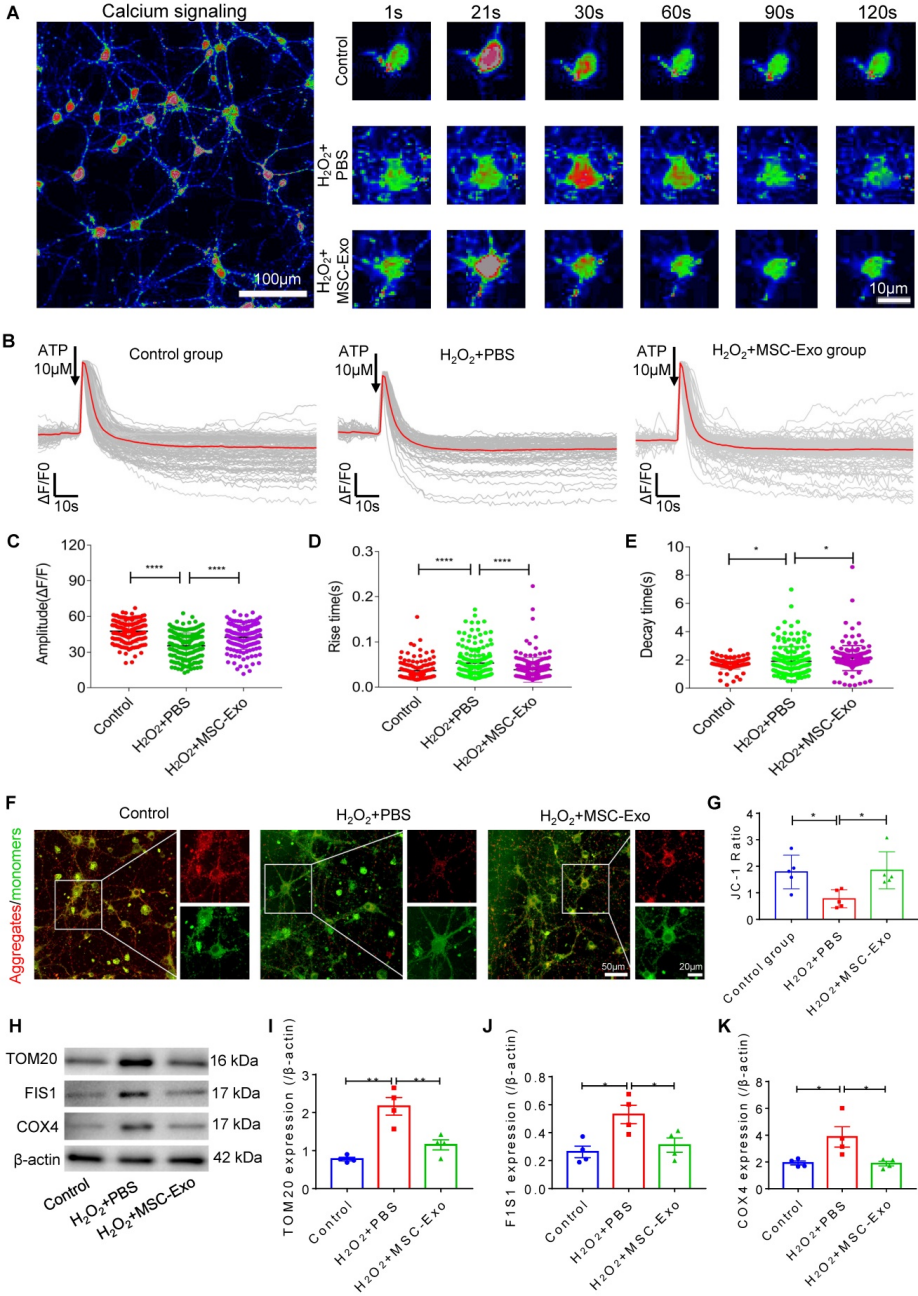
** Amelioration by MSC-EVs on the H_2_O_2_-induced calcium transients and mitochondrial changes in the primary culture.** (A) Representative images show the Fluo-8 AM (a calcium indicator) signaling (left) and fluorescence properties (right, 1~120s) obtained with confocal microscopy in different cultures. Scale bar (left) = 100 µm, scale bar (right) = 10 µm. (B) Igor software assay for fluorescent signals of calcium transients in the first (20s) and second (100s) phases after ATP stimulation in the experimental groups (n > 100 per group). (C-E) Quantification of the amplitude (ΔF/F) (C), rise time (D), and decay time (E) of the intracellular calcium transients in each group (n > 100). (F) JC-1 staining of MMP (red fluorescence represents aggregates; green fluorescence represents monomers) in the primary culture of each group. (G) Histogram shows the ratio of JC-1 fluorescence (Red/Green) in each group (n = 5 per group). Scale bar (G) = 50 µm, bar (H, right images) = 20 µm. (H) Western blotting of TOM20, FIS1, and COX IV expression in different cultures. (I-K) Statistical analysis shows TOM20 (I), FIS1 (J), and COX IV (K) expression in the experimental groups (n = 4 per group). MSC-EVs: mesenchymal stem cell-derived extracellular vesicles; n: number; ATP: adenosine monophosphate; MMP: mitochondrial membrane potential; TOM20: translocase of the outer mitochondrial membrane 20; FIS1: fission 1; COX IV: cytochrome c oxidase IV. * *p* < 0.05, ** *p* < 0.01, **** p < 0.0001.

Since calcium is a ubiquitous intracellular messenger in oxidative stress 29, we used calcium imaging to investigate iron transients in the primary culture of hippocampal neurons. The representative images show altered calcium signaling and fluorescence properties in neurons (Figure 4A). The first-phase calcium response showed a sharp peak, representing a transient, large increase in intracellular calcium after adenosine triphosphate (ATP) stimulation followed by a second-phase response consisting of a slowly declining intracellular calcium concentration in the experimental groups (Figure 4B). Data analysis revealed that, compared to the H_2_O_2_+PBS group, MSC-EV treatment (H_2_O_2_+MSC-EVs) increased the amplitude (ΔF/F) (Figure 4C) and decreased the rate of calcium transients (Figure 4D and E). Moreover, a reduction in the MMP represented by the JC-1 ratio was observed in the H_2_O_2_+PBS group compared with the control treatment (Figure 4G), while the MMP showed improvement in the H_2_O_2_+MSC-EVs treatment group (Figure 4G). Also, compared to the H_2_O_2_+PBS group, decreased protein levels of mitochondrial import receptor subunit TOM20 (Figure 4I), FIS1 (Figure 4J), and COX Ⅳ (Figure 4K) were restored after EV treatment. These results implied that MSC-EVs ameliorated calcium transients and mitochondrial dysfunction in the H_2_O_2_-stimulated primary culture.

### MSC-EVs alleviated seizure-induced oxidative stress in the hippocampus of mice

**Figure 5:**
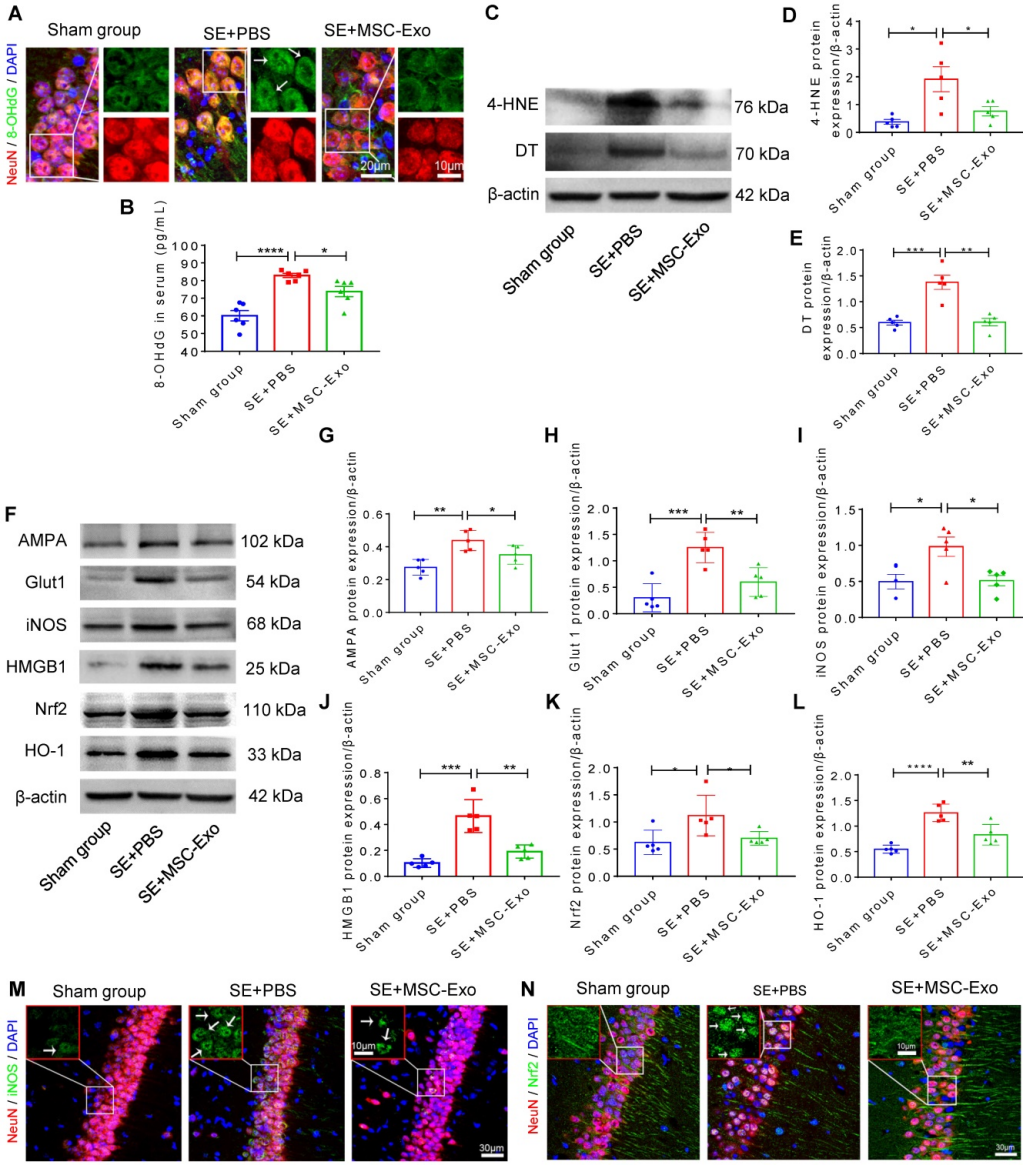
** Antioxidation effect of MSC-EVs on hippocampi of seizure-induced mice.** (A) Representative images show 8-OHdG (green) immunostaining with NeuN (red) and DAPI (blue) in the experimental groups, right magnified images showing the 8-OHdG expression (white arrow, green) in different hippocampal CA1 neurons (red). Scale bar (left) = 20 µm, scale bar (right) = 10 µm. (B) Histogram of the 8- OHdG concentration in each group (n = 6 per group). (C) Western blotting for the lipid (4-HNE) and protein (DT) oxidation in different hippocampal tissues. (D-E) Protein assay for the 4-HNE (D) and DT (E) expression in the experimental groups (n = 5 per group). (F) Western blots of stress-associated molecular patterns in the hippocampus of each group. (G-L) Statistical analysis shows differences in AMPA (G), Glut1 (H), iNOS (I), HMGB1 (J), HO-1 (K), and Nrf2 (L) expression among the experimental groups (n = 5 per group). (M-N) Representative images of oxidative damage and nuclei translocation typified by iNOS (green, M) and Nrf2 (green, N) immunostaining in the hippocampal CA1 neurons (red), respectively, red frames show the magnified images of iNOS (white arrow, green, M) and Nrf2 (whited arrow, green, N) in the experimental group. Scale bars (M, N) = 30 µm, scale bars (upper right magnified images) = 10 µm. MSC-EVs: mesenchymal stem cell-derived extracellular vesicles; n: number; 8-OHdG: 8-hydroxy-2 deoxyguanosin; AMPA: α-amino-3-hydroxy-5-methyl-4-isoxazole-propionic acid; Glut1: glucose transporter 1; iNOS: inducible nitric oxide synthase; HMGB1: high mobility group box 1; HO-1: heme oxygenase-1; Nrf2: nuclear factor erythroid-derived 2, like 2. * *p* < 0.05, ** *p* < 0.01, *** *p* < 0.001, **** *p* < 0.0001.

Oxidative stress is recognized as a critical etiological factor contributing to seizure-induced neuronal damage 12. Therefore, we investigated hippocampal DNA, protein, and lipid oxidation at 4 days post-seizures. Figure 5A displays representative images of DNA damage in the CA1 subfield of hippocampal neurons visualized by immunostaining with 8-OHdG (green) and NeuN (red). Also, ELISA results confirmed the increased concentration of 8-OHdG (*vs*. the Sham group, Figure 5B) in the status epilepticus (SE)+PBS group. Additionally, considerable lipid and protein oxidation, represented by an increase in 4-HNE (Figure 5D) and DT (Figure 5E) expression, respectively, was observed in the SE+PBS group compared to the Sham group. Notably, oxidative characteristics in the hippocampus were significantly reversed in the SE+MSC-EVs group compared to the SE+PBS group (Figure 5A-E). The protein assay showed increased expression of AMPA (a glutamate receptor) (Figure 5G), Glut1 (Figure 5H), iNOS (Figure 5I), HMGB1 (Figure 5J), Nrf2 (Figure 5K) and HO-1 (Figure 5L) in the SE+PBS group compared to the Sham group. Representative images of the hippocampal CA1 subfield also showed iNOS generation (Figure 5M) and nuclear translocation of Nrf2 (Figure 5N) by immunostaining of NeuN (Figure 5M and N) in the SE+PBS group. Remarkably, these molecular alterations were reversed in the EV therapy group (SE+MSC-EVs) compared to the SE+PBS group (Figure 5G-N). These data revealed that MSC-EVs alleviated seizure-induced oxidative response in the hippocampus of mice.

### MSC-EVs restored seizure-induced neuronal morphology alterations and mitochondrial dysfunction in the hippocampus

**Figure 6:**
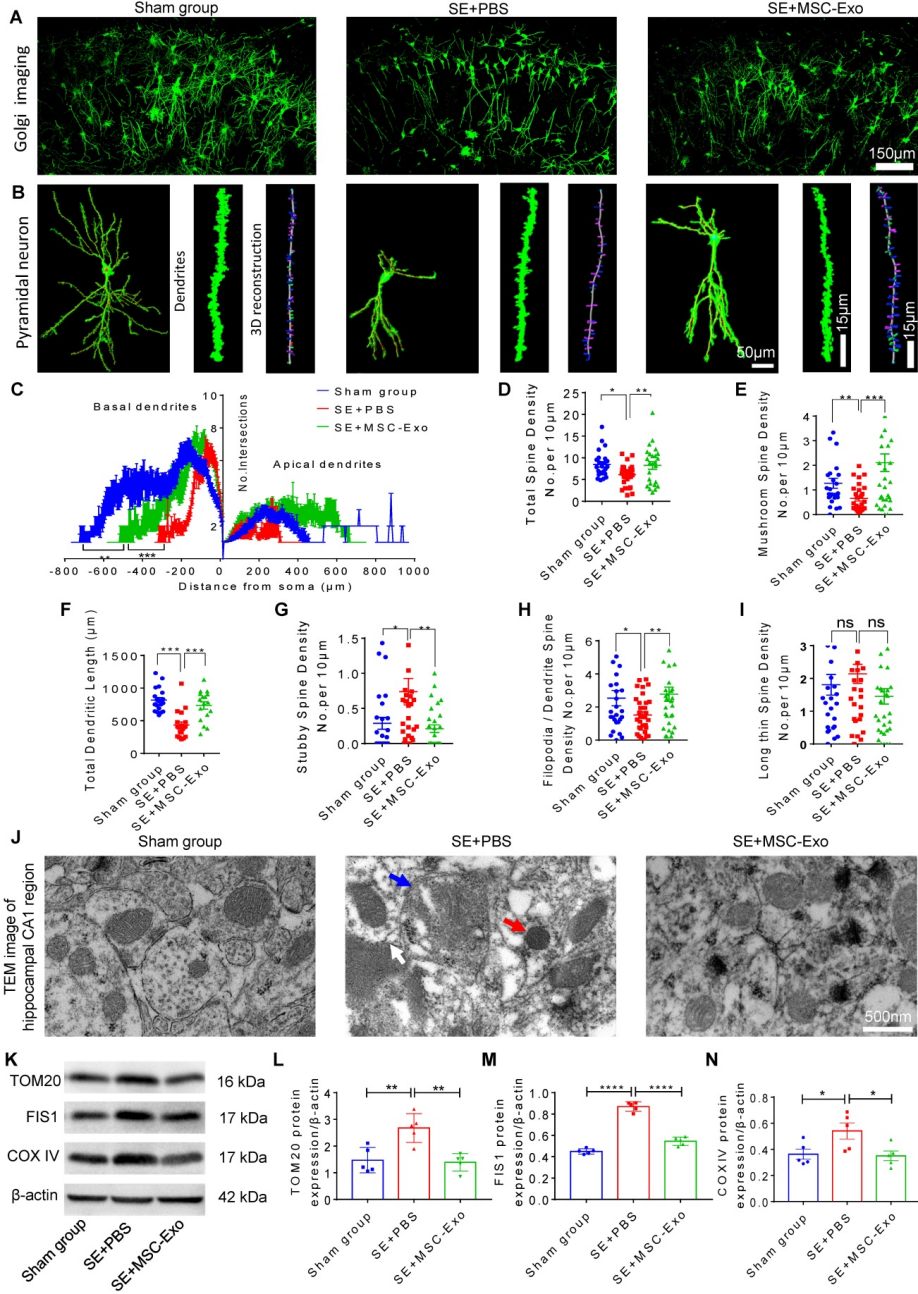
** Restoration of neuronal morphology alterations and mitochondrial changes by MSC-EVs in seizure mice.** (A) Golgi imaging for the neuronal morphology in the experimental groups (n = 5 per group). Scale bar = 150 µm. (B) Representative images show the typical morphology (left), dendrites (middle), and 3D reconstruction of dendrites (right) in hippocampal pyramidal neurons of each group. Scale bar (left) = 50 µm, scale bars (middle, right) = 15 µm. (C) Statistical analysis for the dendritic complexity, including basal dendrites and apical dendrites among the experimental groups. (D-I) Quantification of the total spine density (D), mushroom spine density (E), total dendritic length (F), stubby spine density (G), filopodia/dendrite spine density (H), and long thin spine density (I) in hippocampal CA1 pyramidal neurons of each group. (J) Representative image shows the swelling of mitochondria (blue arrow) and their membrane (white arrow), and increased cristae density (red arrow) in hippocampal CA1 pyramidal neurons of each group. Scale bar = 500 nm. (K) Western blots of mitochondrial damage markers in hippocampal tissues of mice (K). (L-N) Statistical analysis for the TOM20 (L), FIS1 (M), and COX IV expression (N) in experimental groups (n = 5 per group). MSC-EVs: mesenchymal stem cell-derived extracellular vesicles; ns: no significance; n: number; TOM20: translocase of the outer mitochondrial membrane 20; FIS1: fission 1; COX IV: cytochrome c oxidase IV. * *p* < 0.05, ** *p* < 0.01, *** *p* < 0.001, **** *p* < 0.0001.

Hippocampal pyramidal neurons are known to exhibit typical morphological alterations in epileptic models and patients 30, 31. We examined the dendritic processes and spines of pyramidal neurons using Golgi staining at 4 days post-seizures. As displayed in Figure 6, obvious morphological changes were evident among the Sham, SE+PBS, and SE+MSC-EVs groups (Figure 6A). The neuronal phenotype (Figure 6B) analysis showed that structural defects in the dendrites and spines of pyramidal cells were characterized by lower dendritic complexity of basal and apical dendrites (Figure 6C) and lower total and mushroom spine density (Figure 6D) (Figure 6E) in the SE+PBS group than in the Sham mice. Three-dimensional reconstruction of dendrites (Figure 6B) indicated that, compared to the Sham group, seizures reduced the total dendritic length (Figure 6F), stubby spine density (Figure 6G), and filopodia/dendrite spine density (Figure 6H) of hippocampal CA1 pyramidal neurons. Significantly, these structural defects in pyramidal neurons were reversed in the MSC-EVs group (Figure 6C-H) compared to the SE+PBS group. Additionally, seizure or MSC-EVs did not appear to affect the long thin spine density of pyramidal cells (Figure 6I).

Next, we detected mitochondrial changes in hippocampal pyramidal neurons of each group using TEM. As shown in Figure 6J, the SE+PBS group exhibited noticeable mitochondrial ultrastructure changes, such as swelling of mitochondria and their membranes and increased cristae density, compared with the Sham group, while a normal mitochondrial structure was observed in the SE+MSC-EVs group. Moreover, Western blotting (Figure 6K) showed higher expression of TOM20 (Figure 6L), FIS1 (Figure 6M), and COX Ⅳ (Figure 6N) in hippocampal tissues of the SE+PBS group than the Sham group. Notably, these protein changes were decreased by MSC-EV treatment compared to PBS treatment. These results implied that seizure-induced neuronal morphology alterations and mitochondrial dysfunction in the hippocampus could be restored by MSC-EV therapy.

### MSC-EVs ameliorated hippocampal neuron damage-associated sequelae of seizures in mice

**Figure 7:**
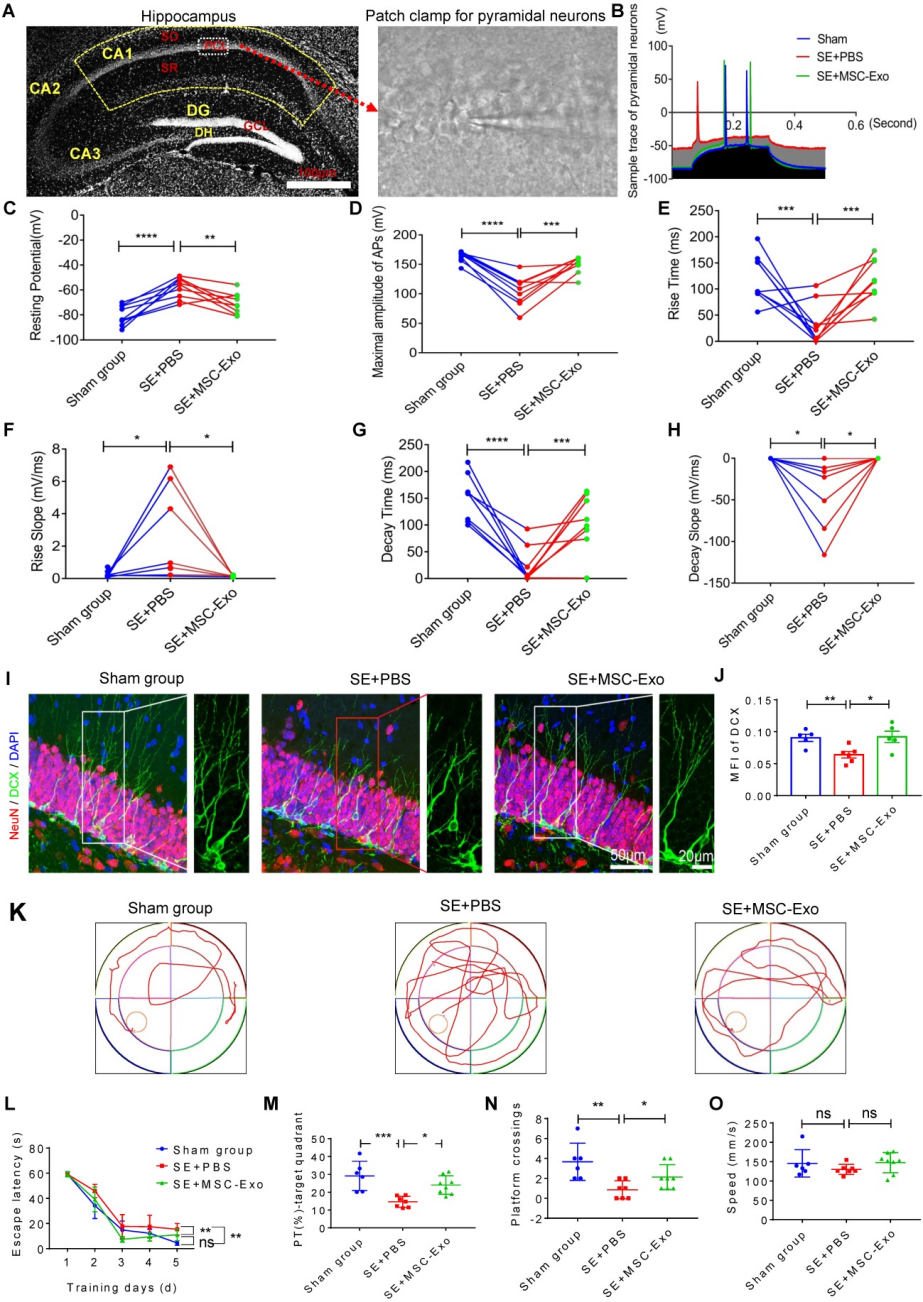
** Restorative effects of MSC-EVs on hippocampal neurons in the chronic stage of seizures.** (A) Representative images show hippocampal subfields (left) and whole-cell patch-clamp for CA1 pyramidal neurons (right). Scale bar = 100 µm. (B) Representative potential traces of pyramidal neurons in each group. (C-H) Statistical analysis of the resting potential (C), maximal amplitude of APs (D), rise time (E), slope (F), decay time (G), and slope (H) in hippocampal CA1 pyramidal neurons of each group (n = 6~9 cells per group). (I) Representative images of DCX (green) immunostaining with NeuN (red) and DAPI (blue) in the hippocampal dentate gyms (DG) of experimental groups, right images show the magnification of DCX (green) staining. Scale bar (left images) = 50 µm, scale bar (right magnified images) = 20 µm. (J) Statistical analysis of the MFI of DCX in the DG of each group (n = 4 per group). (K-O) Representative traces show the MWM test for experimental groups (K), statistical analysis for the escape latency (L), PT (%) target quadrant (M), platform crossings (N) and swim speed (O) in each group (n = 6~8 per group). MSC-EVs: mesenchymal stem cell-derived extracellular vesicles; SO: stratum oriens; PCL: pyramidal cell layer; SR: stratum radiatum; ns: no significance; n: number; DG: dentate gyrus; GCL: granular cell layer; DH: dentate hilus; DCX: doublecortin; DAPI: 4',6-Diamidino-2-phenylindole; DG: dentate gyrus; MFI: mean fluorescence intensity; MWM: Morris water maze. * p < 0.05, ** *p* < 0.01, *** *p* < 0.001, **** p < 0.0001, ns *p* > 0.05.

We used the whole-cell patch-clamp recording to determine the functional reconstruction of hippocampal neurons in seizures and detect MSC-EV effects on membrane properties and excitability in CA1 pyramidal neurons of hippocampal slices (Figure 7A) at 3 months post-seizure. Analysis of the traces of pyramidal neurons (Figure 7B) illustrated marked changes in resting potential (Figure 7C), maximum amplitude of AP (Figure 7D), rise time and slope (Figures 7E & 7F), and decay time and slope (Figures 7G & 7H) in the SE+PBS group compared to the Sham group; these observations were consistent with a previous report 32. Following EV therapy, these neuronal membrane properties and excitability were significantly reversed in the SE+MSC-EV group compared to the SE+PBS group (Figure 7C-H). We also used DCX (a newborn neuronal marker) immunostaining with NeuN to examine neurogenesis in each group (Figure 7I and Figure S5). Compared to the Sham group, the SE+PBS group exhibited abnormal neurites (right magnified image, Figure 7I) and decreased neurogenesis (Figure 7J and Figure S5) in the chronic phase. In contrast, the pattern and extent of neurogenesis observed after MSC-EV administration were equivalent to those in the age-matched Sham group but better than those in the SE+PBS group (Figure 7I, J and Figure S5).

Next, cognitive decline in the experimental groups was detected by the MWM test (Figure 7K). In the hidden platform test, repeated measures of ANOVA showed that the EV-treated mice exhibited a shorter escape latency than the seizure mice that were administered PBS (Figure 7L). Also, in the Sham group, seizures induced a marked decrease in the percent of total swim distance in the target quadrant (PT) (Figure 7M) and platform crossings (Figure 7N). In contrast, these tendencies were markedly elevated in the SE+MSC-EV group. Besides, there was no difference in the swim speed between the groups (Figure 7O). These data suggested that EV therapy facilitated the reconstruction of hippocampal neuronal function in the chronic stage of seizures.

### The Nrf2 defense system was involved in the antioxidative effects of MSC-EVs in seizures

**Figure 8:**
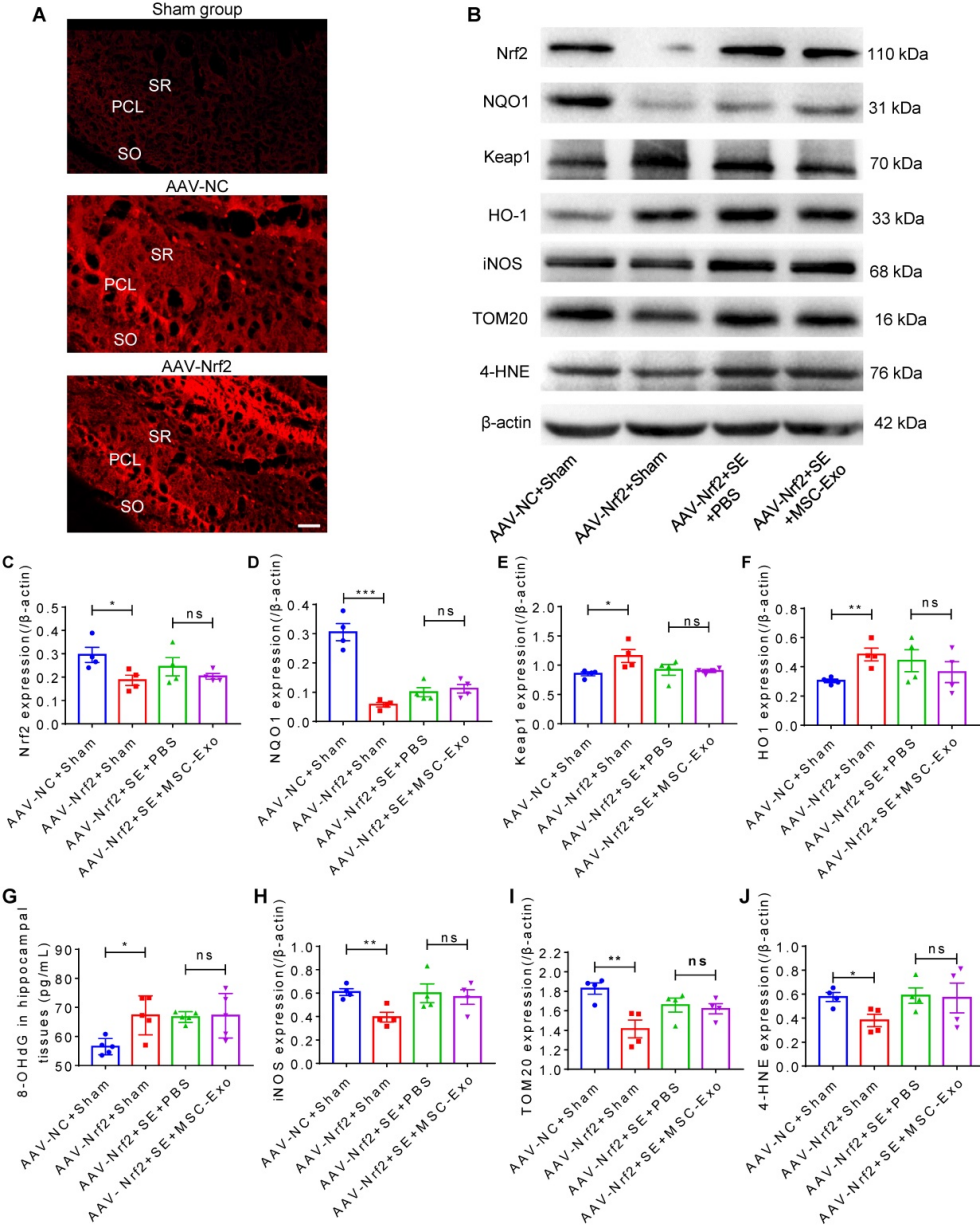
** Nrf2-mediated antioxidant defense system in EV therapy.** (A) Confocal images for the fluorescence properties (red) in hippocampi of experimental groups. Scale bar = 50 µm. (B) Western blots of Nrf2 signaling and typical stress-associated molecular patterns in experimental groups. (C-F) Histograms of thee Nrf2 (C), NQO1 (D), Keap1 (E), HO-1 (F) expression in experimental groups (n = 5 per group). (G) ELISA for the 8-OHdG concentration in the hippocampus of each group (n = 5 per group). (H-J) Protein assay for stress-associated markers iNOS (H), TOM20 (I), and 4-HNE (J) in the experimental groups (n = 4 per group). MSC-EVs: mesenchymal stem cell-derived extracellular vesicles; SO: stratum oriens; PCL: pyramidal cell layer; SR: stratum radiatum; ns: no significance; n: number; Nrf2: nuclear factor erythroid-derived 2, like 2. NQO1: NAD(P)H quinone oxidoreductase 1; Keap1: Kelch-like ECH-associated protein 1; HO-1: heme oxygenase-1; TOM20: translocase of the outer mitochondrial membrane 20; 4-HNE: 4-hydroxynoneal; 8-OHdG: 8-hydroxy-2 deoxyguanosine; iNOS: inducible nitric oxide synthase. * *p* < 0.05, ** *p* < 0.01, *** *p* < 0.001, ns *p* > 0.05.

To further clarify the mechanism underlying the protective effects of EV therapy on seizure-induced oxidative neuronal damage, intrahippocampal (CA1 subfield, Figure 8A) injection of AAV was used to knockdown *Nrf2* expression in the experimental groups. Western blotting (Figure 8B) showed that compared to the NC group, mice treated with AAV-Nrf2 showed a significant decrease in Nrf2 (Figure 8C) and NQO1 (Figure 8D) protein levels and an increase in HO-1 (Figure 8E) and Keap1 (Figure 8F) protein levels in the hippocampus. Although these signals were altered following seizure induction in hippocampal tissues, no significant differences were observed between the AAV-Nrf2+SE+MSC-EV and AAV-Nrf2+SE+PBS groups (Figure 8C-F). ELISA assay also showed that AAV injection (AAV-Nrf2+Sham group) resulted in an oxidative response indicated by increased expression of 8-OHdG (Figure 8G) compared to the AAV-NC+Sham group. Interestingly, compared to the AAV-NC+Sham group, Nrf2 knockdown also resulted in a reduction of iNOS (Figure 8H), TOM20 (Figure 8I), and 4-HNE (Figure 8J) in the AAV-Nrf2+Sham group. However, following AAV-Nrf2+SE+PBS injection, EV therapy (AAV-Nrf2+SE+MSC-EVs) did not reduce the expression of oxidative markers (Figure 8G-J) induced by seizure. Thus, these results indicated that *Nrf2* knockdown attenuated the antioxidant potential of MSC-EVs on seizure-induced damage in the hippocampus, suggesting the involvement of the Nrf2 defense system in the antioxidative effect of MSC-EVs in seizures.

## Discussion

MSC-EVs, as nanotherapeutic agents, have shown great promise in epilepsy treatment due to their immunomodulatory and regenerative properties, but few studies have focused on the underlying mechanisms, such as oxidative stress, for attaining therapeutic efficacy. Here, we determined mechanisms underlying the therapeutic effects of MSC-EVs and clarified their role in restoring hippocampal neuronal structure, function, and molecular patterns damaged by seizures.

Oxidative stress has recently been recognized as a critical etiological factor contributing to seizure-induced neuronal damage 12, 33. In the present study, the primary culture of hippocampal neurons was pretreated with MSC-EVs to induce nanovesicle uptake. Subsequently, the cells were stimulated with H_2_O_2_ to induce oxidative stress typified by `decreased activities of FRAP and multiple antioxidant enzymes (CAT, SOD, and GSH-PX), as well as excess ROS production, as previously described 34-36. Markedly, MSC-EVs demonstrated antioxidant potential in the primary culture by reversing the H_2_O_2_-induced oxidative stress. We studied the antioxidative effect of MSC-EVs four days post-seizure because the hippocampus is susceptible to seizures, and neuronal damage is significant in the early stage 6. Decreased expression of oxidative markers 8-OHdG, 4-HNE, and DT 37, and altered 8-OHdG immunostaining with NeuN in the MSC-EV group compared to the PBS group indicated the remarkable antioxidative effect of the treatment in cultured cells and animal models in alleviating seizure-induced neuronal damage.

EVs contain a large number of functional RNAs, proteins, and lipids, which mediate the antioxidative and immunomodulatory effects in various diseases 4, 38, 39, and the EV-mediated transfer of miRNAs to target cells has been shown to play a crucial role in these effects 39, 40. Therefore, we used miRNA sequencing to identify the potential cargoes responsible for the antioxidative effects of EVs. MSCs preconditioned with H_2_O_2_ were reported to have better antioxidant activity 22. In our study, the conditioned EVs contained a series of upregulated antioxidant miRNAs, including but are not limited to miR-215-5p, miR-424-5p, miR-31-3p, miR-193b-3p and miR-200b-3p. This is consistent with the GO classification of molecular functions, suggesting that exosomal miRNAs are involved in antioxidant activity. Hence, we used miRNA inhibitors to examine the antioxidant potential of the miRNAs on H_2_O_2_-stimulated hippocampal neurons. It is well established that miRNAs or their mimics have a strong regulatory impact on protein expression and gene networks 41. Even though numerous miRNAs (*e.g.,* miR-320, miR-451a, and miR-1202) released from MSC-EVs have been implicated in oxidative stress 40, 42, single or multiple miRNAs from MSC-EVs might not completely recapitulate the antioxidative effects of EVs. Therefore, in the present study, instead of MSC-EV miRNAs, we used MSC-EVs to investigate the therapeutic effects on oxidative neuronal damage.

Oxidative stress-mediated neuronal damage is a hallmark of many neurological disorders, such as seizures 12. Although previous findings suggested that MSCs and MSC-derived EVs could inhibit oxidative injury 21, 40, their efficacy against oxidative neuronal dysfunction and structural damage remains poorly understood. Electrophysiological disturbances typically manifest in neuronal dysfunction, and CA1 pyramidal neurons are particularly susceptible to oxidative stress during epilepsy 14, 43. Specifically, a depolarizing response increases the spontaneous firing and number of APs, while an increment of input resistance causes a lesion of cellular excitability in neurons 44, 45. Remarkably, after EV therapy, improved neuronal membrane properties and excitability suggest that the ability of MSC-EVs to repair hippocampal electrophysiological disturbances in cell and animal models. Besides, hippocampal pyramidal neurons exhibit typical morphological alterations in epileptic models and patients 30, 31. In particular, dendritic spine geometry is critical for molecular expression (*e.g.,* AMPA) and stress resilience in hippocampal CA1 pyramidal neurons 46, 47.

In this study, we have shown that the lower dendritic complexity and spine density reduction were restored by EV treatment, suggesting the reconstructive ability of MSC-EVs on seizure-induced morphological alterations in hippocampal CA1 pyramidal neurons. We also used neuronal apoptosis, a major prototypic form of programmed cell death under oxidative stress 48 to demonstrate neuroprotection of MSC-EVs on the primary culture subjected to H_2_O_2_ stimulation. Abnormal neurogenesis in the hippocampal DG is a frequent sequela of epilepsy that further contributes to seizure development and associated cognitive decline 49, 50. Remarkably, MSC-EV treatment helps maintain a normal pattern and extent of neurogenesis for an extended period and reduces the degree of abnormal neurogenesis in animal models, indicating an improvement of the seizure-induced aberrant neurogenesis in the hippocampal DG subfield. Also, hippocampal neurons and their extended networks contribute to encoding and recall of learning and memory 51. The improved neurobehavior in the MWM test following EV therapy suggested that the disrupted spatial learning and memory can be restored by MSC-EV administration to improve seizures in mice.

Calcium is a ubiquitous intracellular messenger and acts as a key regulator in multiple physiological functions 29. In neurons, the continuous firing of APs leads to calcium cycling processes and abnormal calcium homeostasis occurs due to oxidative stress, resulting in neuronal dysfunction or loss 29, 52. We used calcium imaging to investigate the iron transients in the primary culture and observed increased amplitude and a faster change in the response, suggesting that MSC-EVs ameliorated H_2_O_2_ stimulation-induced calcium transients in the primary culture of hippocampal neurons. Moreover, mitochondria act as local calcium buffers to adjacent calcium release sites and are essential organelles in calcium homeostasis control 53-55. In the present study, the normalized mitochondrial ultrastructure detected by TEM in the MSC-EV group revealed that EV treatment prevented neuronal mitochondrial alterations in response to seizure insults. Furthermore, mitochondria exist in dynamic networks that undergo fusion and fission, the perturbation of which can contribute to mitochondrial dysfunction underlying many aspects of neurodegenerative changes 56-58. Notably, reduced expression of TOM20, FIS1, and COX Ⅳ in the EV-treated group indicated that MSC-EVs restored mitochondrial fission/fusion and respiratory chain when exposed to oxidative insults. Given that calcium signaling and mitochondrial alterations are critical for maintaining the stability of neuronal function 56, 57, our results demonstrated that MSC-EVs ameliorated oxidative stress-induced neuronal dysfunction* in vitro* and *in vivo*.

SAMPs, including iNOS, HMGB1, HO-1, and Nrf2, are intrinsically involved in oxidative responses and cell homeostasis 59-61. MSC-EV treatment of the primary culture resulted in SAMPs amelioration, implying the therapeutic efficacy of MSC-EVs for the H_2_O_2_ stimulation-induced oxidative neuronal damage. Besides, ROS levels are under the control of ionotropic and metabotropic glutamate receptors (*e.g.* AMPA), and Glut1 is also implicated in neuronal death during cell stress 62, 63. After EV treatment, the reversal of expression of these molecular patterns (SAMPs, AMPA, and Glut1) suggested that MSC-EVs alleviated seizure-induced oxidative stress in the hippocampus of mice. It has been reported that Nrf2-mediated therapies drive neuroprotective effects in epilepsy and other neurological disorders in response to oxidative stress 15, 64. We observed marked variations in Nrf2 and its target gene *HO-1*, as well as other SAMPs between the stress models and the MSC-EV-treated group *in vitro* and *in vivo*, indicating that the Nrf2 signaling pathway might be involved in the antioxidative effects of MSC-EVs in seizures. Thus, AAV injection was used for knocking down *Nrf2* expression to probe the molecular mechanism in the experimental groups. Under physiological conditions, Nrf2 is tethered to the regulatory protein Keap1 in the cytoplasm. However, oxidative stress leads to the nuclear translocation of Nrf2, where it binds to the antioxidant response element in promoters of the target genes *NQO1* and *HO-1* in seizures or other neurological disorders 15, 65. We, therefore, examined these signals, including Nrf2, NQO1, HO-1, and Keap1, as well as the typical oxidative stress markers (8-OHdG, iNOS, TOM20, 4-HNE, and DT) in animal models. The altered protein expression between AAV-NC+Sham and AAV-Nrf2+Sham groups indicated the influence of *Nrf2* knockdown on its target genes. However, no significant differences between the AAV-Nrf2+SE+MSC-EVs and AAV-Nrf2+SE+PBS groups for these signals were detected, suggesting that Nrf2 knockdown attenuated the signaling response to seizure insults in EV therapy.

Consistent with previous reports 64, 65, we also found that Nrf2 knockdown resulted in the neuronal damage indicated by an increase in the 8-OHdG expression compared to the AAV-NC+Sham group. Previous data suggested an unpredictable regulatory role of Nrf2 in stress conditions, whereas adaptive activation of Nrf2 is known to show a protective effect in oxidative stress and diseases 21, 66. However, in our study, the oxidative marker expression did not show significant changes between AAV-Nrf2+SE+MSC-EVs and AAV-Nrf2+SE+PBS groups, indicating that AAV injection attenuated the therapeutic efficacy of MSC-EVs on seizure-induced damage in the hippocampus. These results suggested that the Nrf2 defense system was involved in the antioxidative effect of MSC-EVs in seizures.

In summary, the present study demonstrated that MSC-EVs, as nanotherapeutic agents, exhibited robust antioxidant activity in oxidative stress cell and animal models. Also, oxidative stress-induced neuronal dysfunction, structure alterations, and molecular changes could be restored by EV-therapy *in vitro* and *in vivo*. Additionally, the Nrf2 defense system was involved in the antioxidative effects of MSC-EVs in seizures. These results suggested that the antioxidant activity of MSC-EVs facilitates the restoration of hippocampal neurons after oxidative stress, highlighting the clinical value of exosomal antioxidant activity in seizures.

## Supplementary Material

Supplementary figures and tables.

## Abbreviations

4-HNE: 4-hydroxynoneal
8-OHdG: 8-hydroxy-2 deoxyguanosine
AAV: adeno-associated virus
ACSF: artificial cerebrospinal fluid
PBS: phosphate buffer saline
AMPA: α-amino-3-hydroxy-5-methyl-4-isoxazole-propionic acid
ANOVA: analyzed using one-way analyses of variance
APs: action potentials
ATP: adenosine monophosphate
CAT: catalase
CCM: complete culture medium
CCK-8: Cell Counting Kit-8
COX Ⅳ: cytochrome c oxidase Ⅳ
DAPI: 4',6-Diamidino-2-phenylindole
DCX: doublecortin
DG: dentate gyrus
DH: dentate hilus
DMEM: Dulbecco's modified Eagle's medium
DT: dityrosine
EVs: extracellular vesicles
EGTA: ethylene glycol bis (2-aminoethyl ether) tetraacetic acid
FBS: fetal bovine serum
FRAP: Ferric ion reducing antioxidant power
FIS1: fission 1
GCL: granular cell layer
Glut1: glucose transporter 1
GO: gene ontology
GSH-PX: glutathione peroxidase
HEPES: hydroxyethyl piperazine ethane sulfonic acid
HO-1: heme oxygenase-1
HMGB1: high mobility group box 1
iNOS: inducible nitric oxide synthase
Keap1: Kelch-like ECH-associated protein 1
MFI: mean fluorescence intensity
miRNAs: microRNAs
MMP: mitochondrial membrane potential
MSCs: mesenchymal stem cells
MSC-EVs: mesenchymal stem cell-derived extracellular vesicles
MWM: Morris water maze
NQO1: NAD(P)H quinone oxidoreductase 1
Nrf2: nuclear factor erythroid-derived 2, like 2
NTA: nanoparticle tracking analysis
OD: optical density
PCL: pyramidal cell layer
PVDF: polyvinylidene fluoride
RIPA: radioimmunoprecipitation assay
ROS: reactive oxygen species
SE: status epilepticus
SAMPs: stress-associated molecular patterns
SO: stratum oriens
SOD: superoxide dismutase
SR: stratum radiatum
TBST: Tris-buffered saline Tween
TEM: transmission electron microscope
TOM20: translocase of the outer mitochondrial membrane 20
TSG101: tumor susceptibility gene 101
