# Supplementary material for: Antioxidant activity of mesenchymal stem cell-derived extracellular vesicles restores hippocampal neurons following seizure damage
Source: Theranostics. 2021 Apr 3;11(12):5986–6005. doi: 10.7150/thno.58632 (PMC8058724; doi:10.7150/thno.58632)
Supplement: Supplementary file 1 — Supplementary figures and tables. [file thnov11p5986s1.pdf]

Supplementary Materials

Figure S1. The timeline of the experiments.

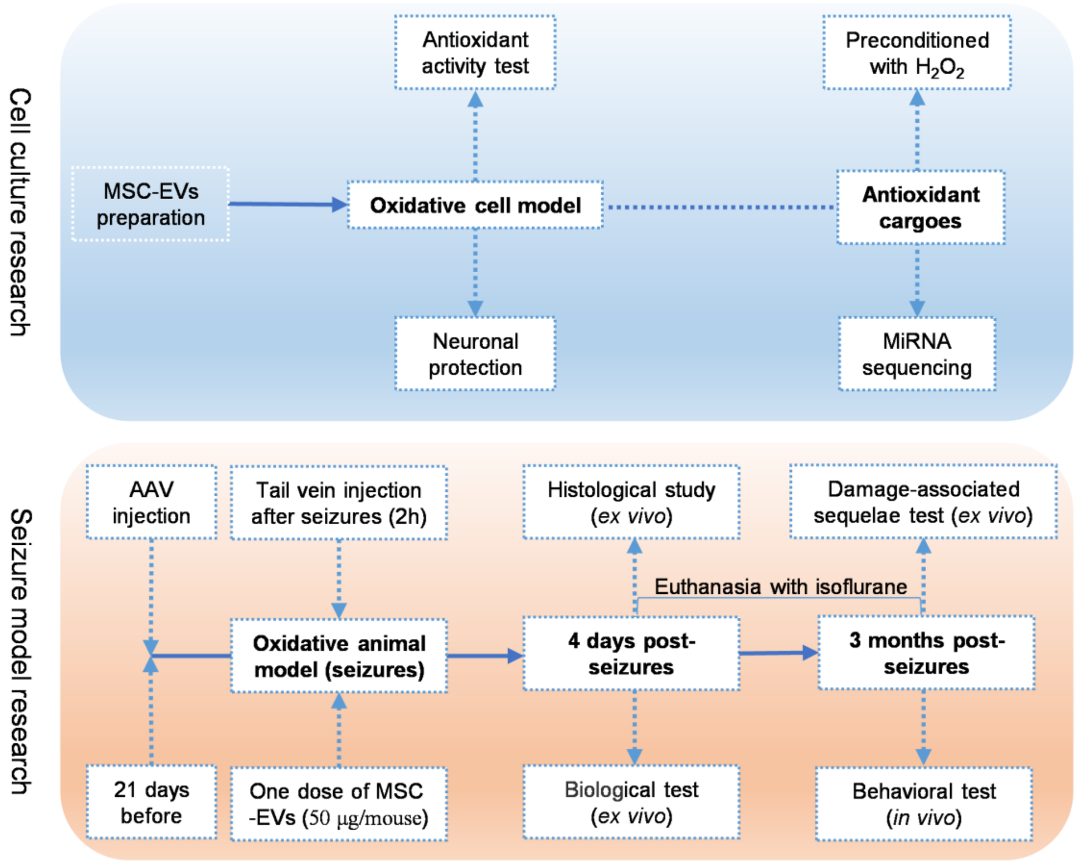

Figure S2. Characterization of MSCs and their derived EVs.

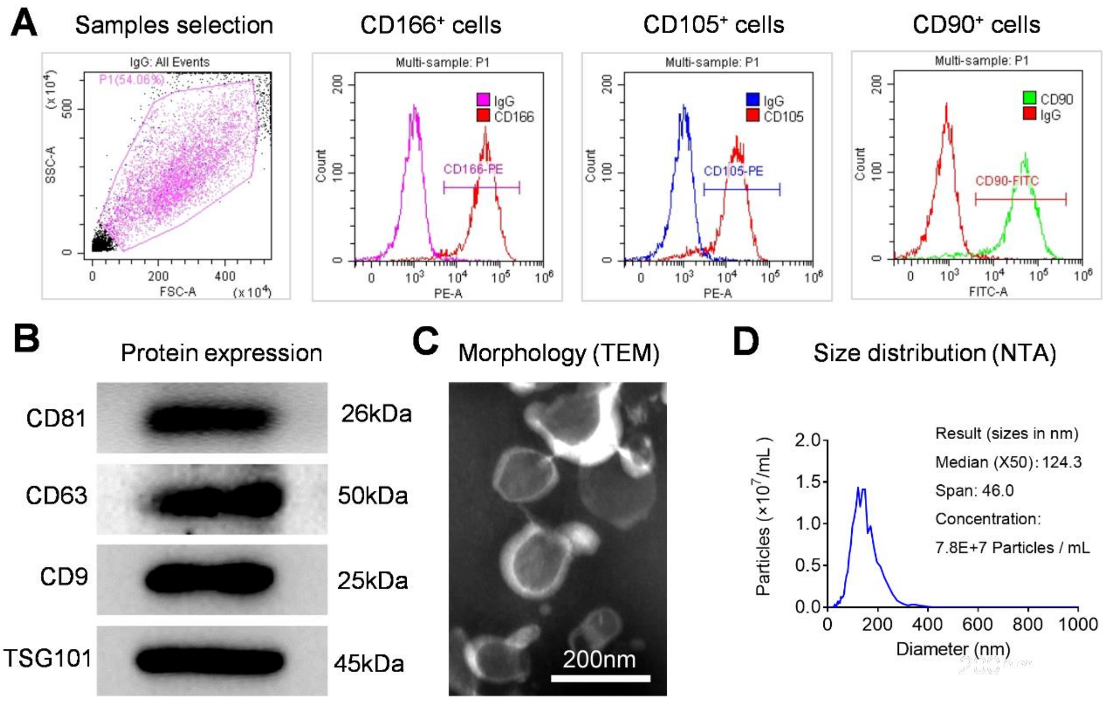

(A) Flow cytometry assay for the mesenchymal marker CD166, CD105 and CD90 in human umbilical cord MSCs. (B) Western blots of classical exosomal marker CD81, CD63, CD9, and TSG101 found on the surface of MSCs. (C) Transmission electron microscope (TEM) image of the exosomal morphology in MSC-EVs. (D) Nanoparticle tracking analysis (NTA) for the size distribution of MSC-EVs, median diameter shown 124.3 nm. Scale bar (C) = 200 nm.

Figure S3. Dose response of MSC-EVs on H_2O_2-stimulated hippocampal neurons.

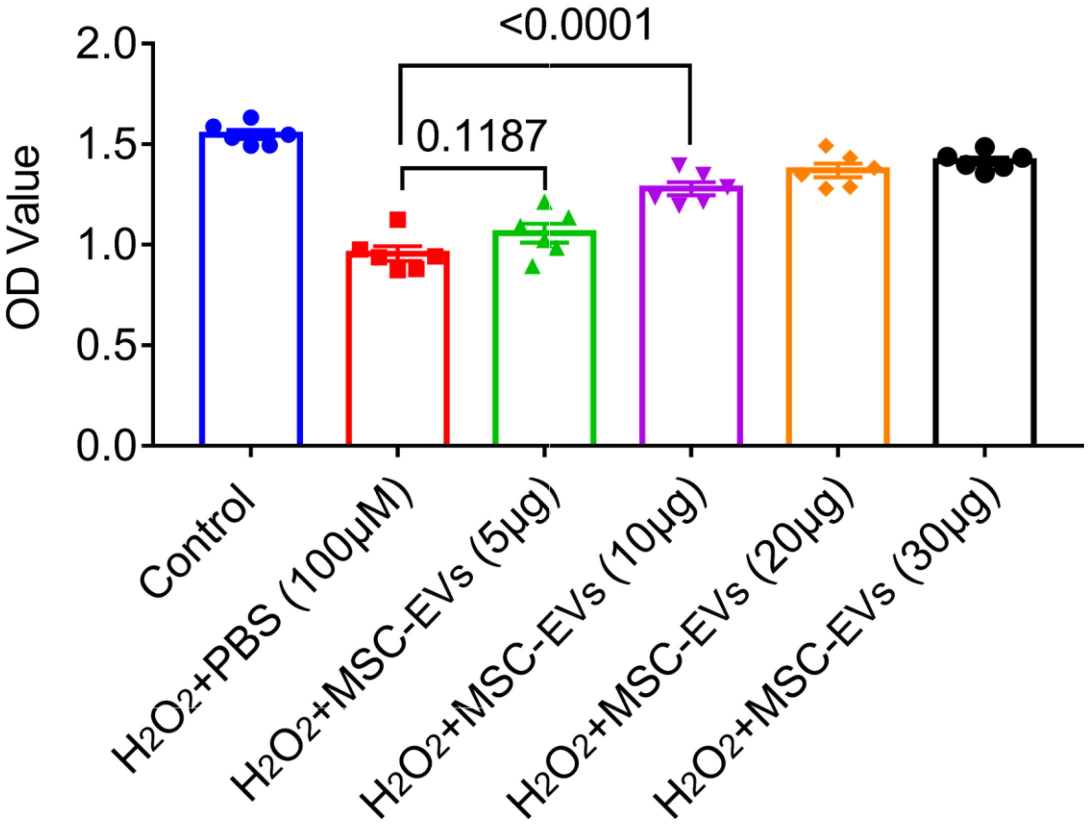

CCK-8 assay shows the cell viability in H_2O_2-stimulated primary culture of hippocampal neurons administered by different dose of MSC-EVs (n = 6 per group). Note a significant increase of optical density (OD) value in 10 μg/ml MSC-EVs treated group in comparison to H_2O_2+PBS group, *p* values were determined by one way-ANOVA.

Figure S4. Dose response of H_2O_2 on primary culture hippocampal neurons.

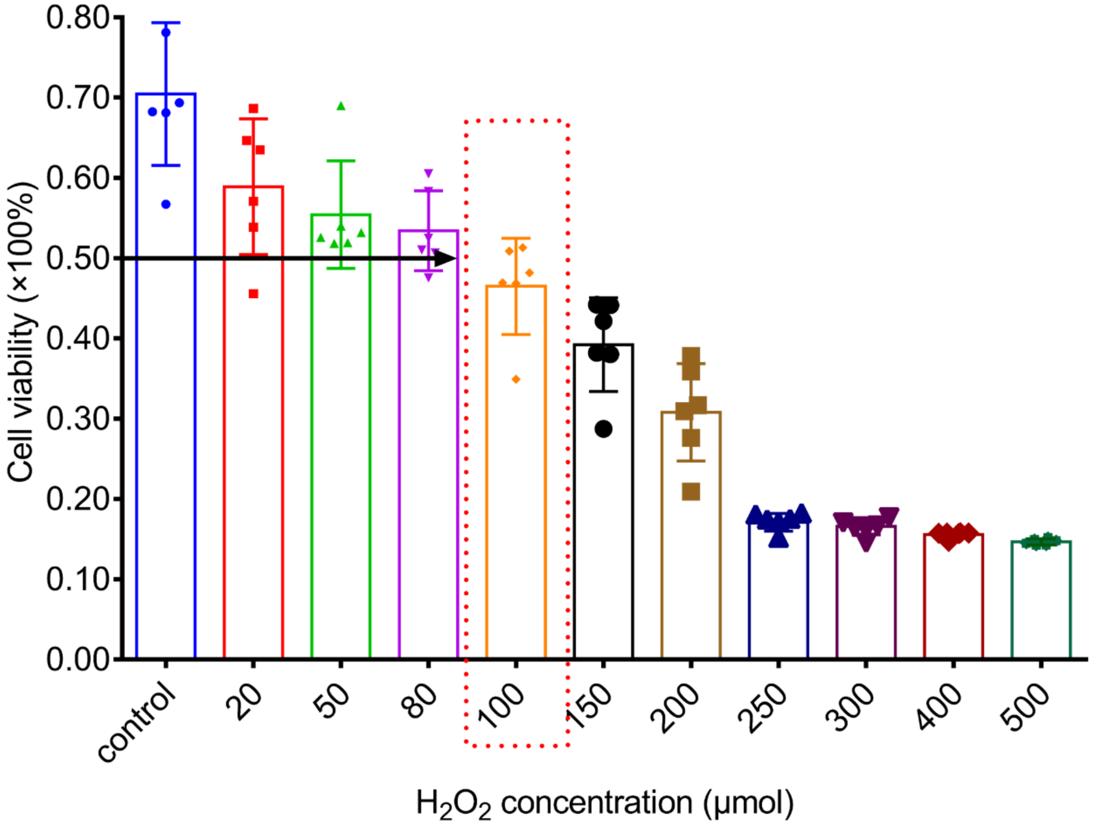

Histogram of different concentrations of H_2O_2-induced cytotoxicity on the primary culture of hippocampal neurons (n = 6 per group) at 3 hours, and the concentration (black arrow, 100 μmol) was used in this study according to the cell viability reduced 50 % in response to H_2O_2 stimulation.

Figure S5. Neurogenesis of hippocampal DG immunostaining for DCX and NeuN.

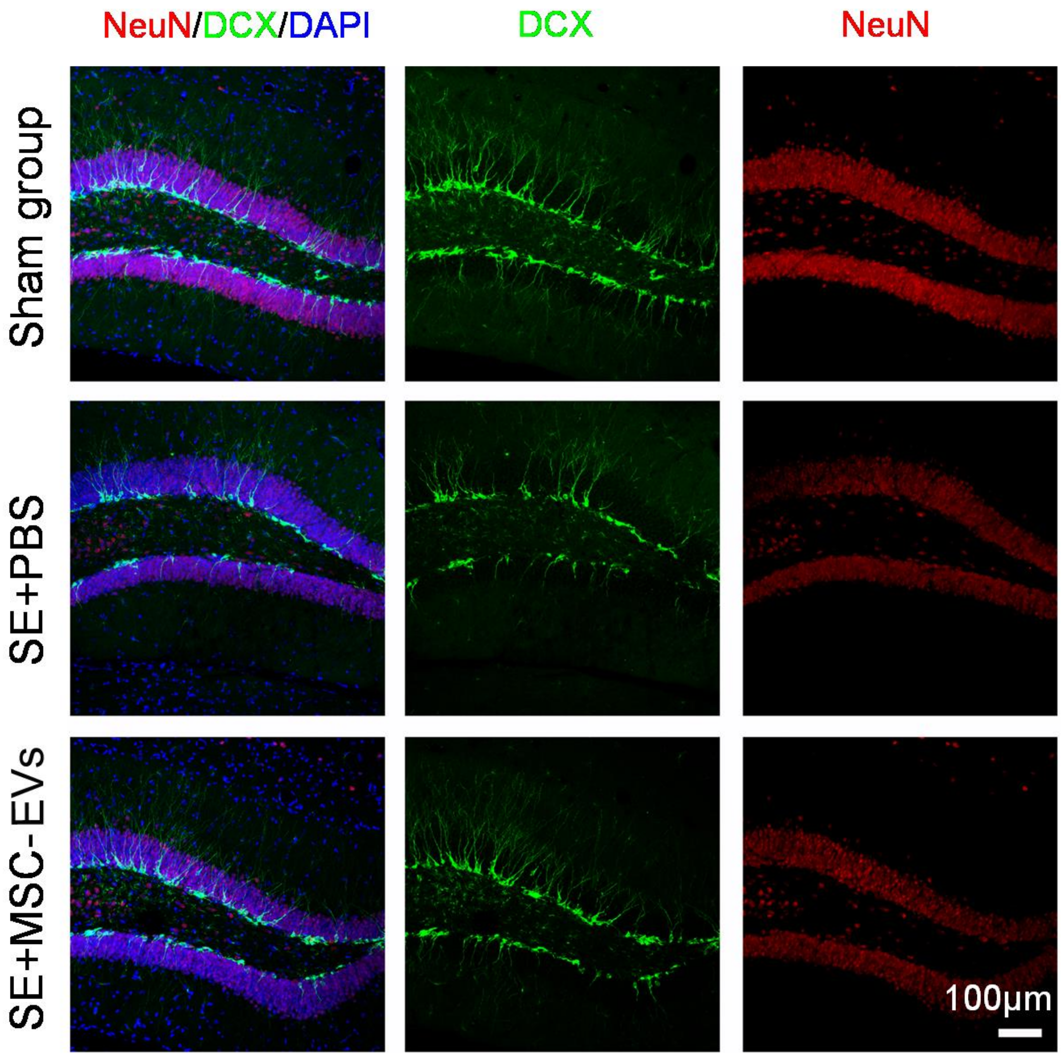

Immunostaining for hippocampal dentate gyrus (DG) by using doublecortin (DCX, a newborn neuronal marker) (green), NeuN (red) and 2-(4-Amidinophenyl)-6-indolecarbamidine dihydrochloride (DAPI) (blue), the representative images showed that, compared to Sham group, animals in SE+PBS group exhibited aberrant neurites (green) and decreased neurogenesis (Figure 7J) in the chronic phase, and MSC-EVs therapy (SE+MSC-EVs) displayed a pattern and extent of neurogenesis that was equivalent to Sham group and a greater extent of neurogenesis than animals in the SE+PBS group (Figure 7J). Scale bar = 100 μm.

Figure S6. Antioxidant response of MSC-EVs on seizure-induced hippocampus.

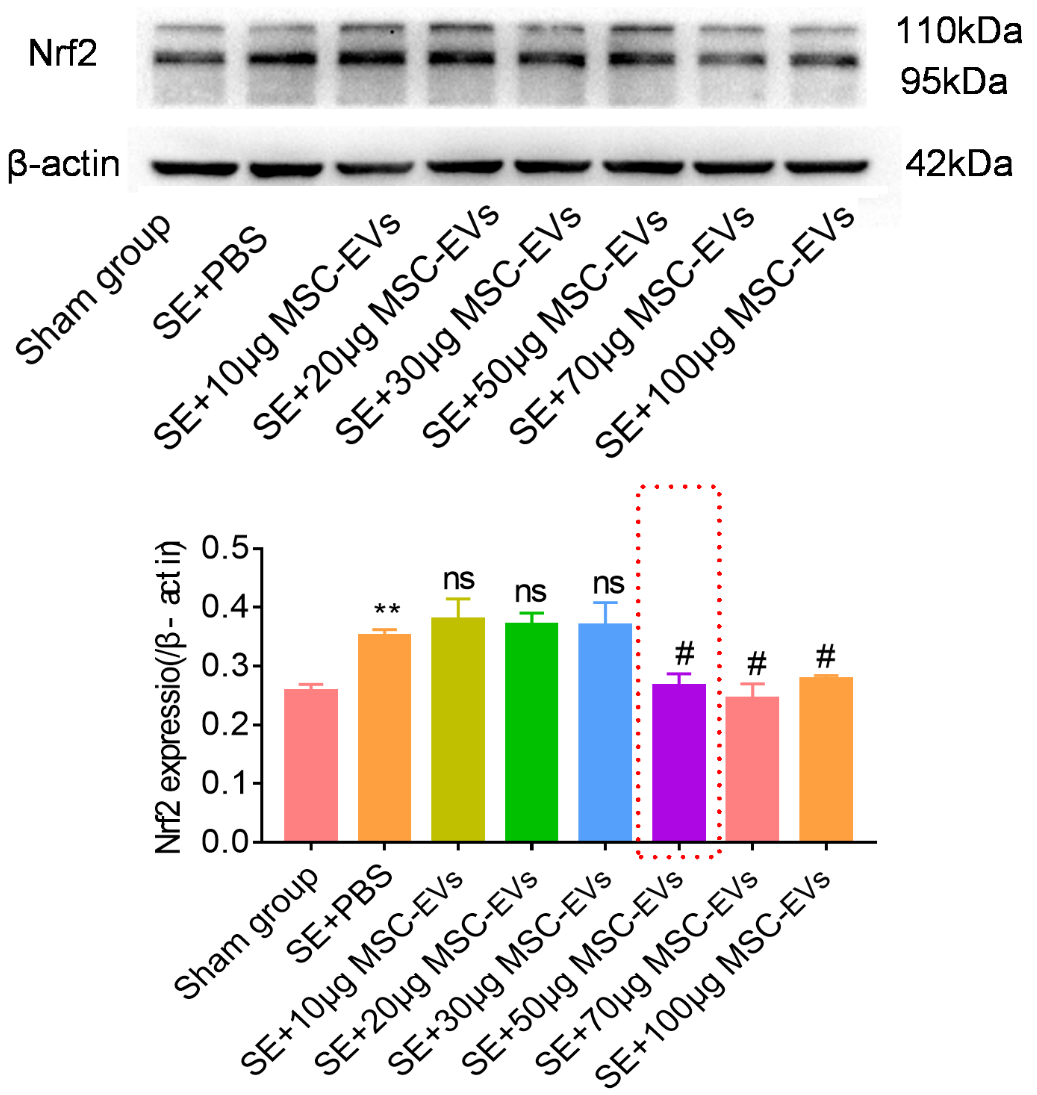

(A) Different dose of MSC-EVs was used to treat seizure mice by tail vein injection, and Western blots of nuclear factor erythroid-derived 2, like 2 (Nrf2, a key antioxidant mediator) expression in hippocampus. (B) Histogram shows the dose response of MSC-EVs on seizure induced Nrf2 expression, the minimal effective concentration (50 μg MSC-EVs per mouse) was employed in this study. ns: no significance. $^{**}p < 0.01$ (*vs*. Sham group), $^{ns}p > 0.05$ (*vs*. SE+PBS), $^{\#}p < 0.05$ (*vs*. SE+PBS).

Table S1. Antioxidant miRNAs in MSC-EVs

miRNA_ID	Log2 fold change	*P* value
hsa-miR-215-5p	6.534131	2.27E-05
hsa-miR-424-5p	6.103048	1.27E-09
hsa-miR-31-3p	6.000901	1.22E-15
hsa-miR-193b-3p	5.519835	5.38E-17
hsa-miR-200b-3p	5.168457	0.000155
hsa-miR-194-5p	5.086709	4.22E-05
hsa-miR-154-3p	4.875326	4.40E-05
hsa-miR-655-3p	4.802787	8.14E-07
hsa-miR-375	4.713645	0.007219
hsa-miR-200a-3p	4.655501	0.003356
hsa-miR-192-5p	4.614421	6.49E-05
hsa-miR-324-5p	4.541701	0.0008
hsa-miR-98-3p	4.423354	0.000753
hsa-miR-7-1-3p	4.408682	0.000581
hsa-miR-30b-5p	4.387363	2.79E-08
hsa-miR-154-5p	4.362341	7.56E-07
hsa-let-7i-3p	4.238247	0.006326
hsa-miR-107	4.174289	5.52E-06
hsa-miR-487a-3p	4.152337	4.71E-05
hsa-miR-93-3p	3.972407	0.004054
hsa-miR-505-3p	3.880051	0.000914
hsa-miR-1185-1-3p	3.864586	2.85E-05
hsa-miR-542-3p	3.761738	0.001316
hsa-miR-454-3p	3.63754	5.07E-05
hsa-miR-34a-5p	3.524039	5.85E-09
hsa-miR-29a-3p	3.496435	3.69E-17
hsa-miR-503-5p	3.440931	4.33E-09
hsa-miR-15b-5p	3.438022	5.80E-08
hsa-miR-500a-3p	3.36699	0.006836
hsa-miR-376c-3p	3.313795	5.80E-07

This Table Sshows the 30 most abundant miRNAs in conditioned MSC-EVs, and the first 5 miRNAs (miR-215-5p, miR-424-5p, miR-31-3p, miR-193b-3p and miR-200b-3p) were selected in the present study.

Table S2. Molecular function of exosomal target genes.

GO_ID	Molecular function	Gene list (n = 58)
0016209	antioxidant activity	ENSG00000120942(UBIAD1),ENSG00000167419(LPO),ENSG00000110244(APOA4),ENSG00000167693(NXN),ENSG00000164938(TP53INP1),ENSG00000198431(TXNRD1),ENSG00000005381(MPO),ENSG00000181019(NQO1),ENSG00000065621(GSTO2),ENSG00000009765(IYD),ENSG00000244734(HBB),ENSG00000073756(PTGS2),ENSG00000074706(IPCEF1),ENSG00000167815(PRDX2),ENSG00000163586(FABP1),ENSG00000143198(MGST3),ENSG00000137857(DUOX1),ENSG00000130508(PXDN),ENSG00000117592(PRDX6),ENSG00000233276(GPX1),ENSG00000121691(CAT),ENSG00000122378(FAM213A),ENSG00000112096(SOD2),ENSG00000131871(VIMP),ENSG00000161544(CYGB),ENSG00000173992(CCS),ENSG00000164294(GPX8),ENSG00000213316(LTC4S),ENSG00000178980(SEPW1),ENSG00000155962(CLIC2),ENSG00000087250(MT3),ENSG00000104687(GSR),ENSG00000206172(HBA1),ENSG00000109610(SOD3),ENSG00000095303(PTGS1),ENSG00000116157(GPX7),ENSG00000123131(PRDX4),ENSG00000211445(GPX3),ENSG00000163631(ALB),ENSG00000129235(TXNDC17),ENSG00000121053(EPX),ENSG00000197448(GSTK1),ENSG00000167468(GPX4),ENSG00000204444(APOM),ENSG00000130203(APOE),ENSG00000271303(SRXN1),ENSG00000176153(GPX2),ENSG00000008394(MGST1),ENSG00000140279(DUOX2),ENSG00000184470(TXNRD2),ENSG00000257017(HP),ENSG00000168454(TXNDC2),ENSG00000115705(TPO),ENSG00000197763(TXNRD3),ENSG00000100577(GSTZ1),ENSG00000120733(KDM3B),ENSG00000198704(GPX6),ENSG00000085871(MGST2)

Table S3. Reagents specificity

Product	Catalog	Manufacturer	Place of Origin
StemPro® osteogenesis	A1007201	Gibco	USA
StemPro® chondrogenesis	A1007101	Gibco	USA
StemPro® adipogenesis	A1007001	Gibco	USA
FBS	10099-141C	Gibco	USA
DMEM/F12	31331093	Thermo Fisher	USA
DMEM	22320030	Thermo Fisher	USA
Neurobasal medium	21103-049	Gibco	USA
B27	17504-044	Gibco	USA
penicillin-streptomycin	15140-122	Gibco	USA
L-glutamine	25030081	Gibco	USA
Cell Counting Kit-8	C0040	Beyotime	China
pilocarpine hydrochloride	S4231	Selleck Chemicals	USA
FRAP	A015-3	Nanjing Jiancheng	China
CAT	S0051	Beyotime	China
SOD	A001-3	Nanjing Jiancheng	China
GSH-PX	A005	Nanjing Jiancheng	China
2′,7′-dichlorodihydro fluoroscein diacetate	D6883	Sigma-Aldrich	USA
Annexin-V/PI	556547	BD	USA
miRNeasy Serum/Plasma Kit	217184	Qiagen	Germany
8-OHdG	CEA660Ge	Cloud-Clone	China
Fluo-8 AM	ab142773	Abcam	USA
JC-1	3520-43-2	Solarbio	China
DAPI	D9542	Sigma-Aldrich	USA
RIPA lysis buffer	P0013B	Beyotime	China
Lipofectamine 3000	L3000015	Invitrogen	USA

Table S4. MiRNA sequences

Isotype	Sequences
miRNA inhibitor NC	CAGUACUUUUGUGUAGUACAA
hsa-miR-215-5p mimics	AUGACCUAUGAAUUGACAGAC
hsa-miR-424-5p mimics	CAGCAGCAAUUCAUGUUUUGAA
hsa-miR-31-3p mimics	UGCUAUGCCAACAUAUUGCCAU
hsa-miR-193b-3p mimics	AACUGGCCCUCAAAGUCCCGCU
hsa-miR-200b-3p mimics	UAAUACUGCCUGGUAAUGAUGA
hsa-miR-215-5p inhibitor	GUCUGUCAAUUCAUAGGUCAU
has-miR-424-5p inhibitor	UUCAAAACAUGAAUUGCUGCUG
has-miR-31-3p inhibitor	AUGGCAAUAUGUUGGCAUAGCA
has-miR-193b-3p inhibitor	AGCGGGACUUUGAGGGCCAGUU
has-miR-200b-3p inhibitor	UCAUCAUUACCAGGCAGUAUUA
miRNA inhibitor NC-FAM	CAGUACUUUUGUGUAGUACAA

Table S5. Primary antibodies

Isotype	Catalog	Manufacturer	Place of Origin	Concentration
CD166	559263	BD Biosciences	USA	1:200
CD105	560839	BD Biosciences	USA	1:200
CD90	555595	BD Biosciences	USA	1:200
CD45	555483	BD Biosciences	USA	1:200
CD34	550761	BD Biosciences	USA	1:200
CD11b	555388	BD Biosciences	USA	1:200
CD81	10630D	Invitrogen	USA	1:1000
CD63	10628D	Invitrogen	USA	1:1000
CD9	ab92726	Abcam	USA	1:1000
TSG101	ab125011	Abcam	USA	1:1000
8-OHdG	AB5830	Millipore	USA	1:200
NeuN	ABN78	Millipore	USA	1:1000
DCX	sc-271390	Santa Cruz	USA	1:200
NeuN	ab104224	Abcam	USA	1:1000
iNOS	18985-1-AP	Proteintech	USA	1:100
Nrf2	ab31163	Abcam	USA	1:100
Nrf2	16396-1-AP	Proteintech	USA	1:1000
HO-1	10701-1-AP	Proteintech	USA	1:1000
Keap1	10503-2-AP	Proteintech	USA	1:1000
4-HNE	MA5-27570	Invitrogen	USA	1:1000
DT	MA5-27575	Invitrogen	USA	1:1000
HMGB1	10829-1-AP	Proteintech	USA	1:1000
TOM20	11802-1-AP	Proteintech	USA	1:5000
FIS1	10956-1-AP	Proteintech	USA	1:1000
COX IV	11242-1-AP	Proteintech	USA	1:5000
AMPA	ab109450	Abcam	USA	1: 2000
Glut1	21829-1-AP	Proteintech	USA	1:1000
β-actin	AC026	Abclonal	China	1:10000

Table S6. Secondary antibodies

Isotype	Catalog	Manufacturer	Origin	Concentration
Alexa Fluor 488-conjugated donkey anti-mouse IgG (H+L)	A-21202	Invitrogen	USA	1:200
Alexa Fluor 488-conjugated donkey anti-rabbit IgG (H+L)	A-21206	Invitrogen	USA	1:200
Alexa Fluor-conjugated 488 donkey anti-goat IgG (H+L)	A-11055	Invitrogen	USA	1:200
Alexa Fluor 594-conjugated donkey anti-mouse IgG (H+L)	A-21203	Invitrogen	USA	1:200
Alexa Fluor 594-conjugated donkey anti-rabbit IgG (H+L)	A-21207	Invitrogen	USA	1:200
horseradish peroxidase-conjugated goat anti-rabbit	31460	Thermo Fisher	USA	1:25000
horseradish peroxidase-conjugated goat anti-mouse	31430	Thermo Fisher	USA	1:50000
